# Development and Validation of a Six-Gene Prognostic Signature for Bladder Cancer

**DOI:** 10.3389/fgene.2021.758612

**Published:** 2021-12-06

**Authors:** Fei Xu, Qianqian Tang, Yejinpeng Wang, Gang Wang, Kaiyu Qian, Lingao Ju, Yu Xiao

**Affiliations:** ^1^ Department of Laboratory Medicine, Zhongnan Hospital of Wuhan University, Wuhan, China; ^2^ Department of Breast and Thyroid Surgery, Zhongnan Hospital of Wuhan University, Wuhan, China; ^3^ Laboratory of Precision Medicine, Zhongnan Hospital of Wuhan University, Wuhan, China; ^4^ Department of Biological Repositories, Zhongnan Hospital of Wuhan University, Wuhan, China; ^5^ Human Genetic Resource Preservation Center of Hubei Province, Wuhan, China; ^6^ Human Genetic Resource Preservation Center of Wuhan University, Wuhan, China

**Keywords:** bladder cancer, biomarkers, cancer-specific survival, six-gene prognostic signature, bioinformatics analysis

## Abstract

Human bladder cancer (BCa) is the most common urogenital system malignancy. Patients with BCa have limited treatment efficacy in clinical practice. Novel biomarkers could provide more crucial information conferring to cancer diagnosis, treatment, and prognosis. Here, we aimed to explore and identify novel biomarkers associated with cancer-specific survival of patients with BCa to build a prognostic signature. Based on univariate Cox regression, Lasso regression, and multivariate Cox regression analysis, we conducted an integrated analysis in the training set (GSE32894) and established a six-gene signature to predict the cancer-specific survival for human BCa. The six genes were Cyclin Dependent Kinase 4 (*CDK4*), E2F Transcription Factor 7 (*E2F7*), Collagen Type XI Alpha 1 Chain (*COL11A1*), Bradykinin Receptor B2 (*BDKRB2*), Yip1 Interacting Factor Homolog B (*YIF1B*), and Zinc Finger Protein 415 (*ZNF415*). Then, we validated the prognostic value of the model by using two other datasets (GSE13507 and TCGA). Also, we conducted univariate and multivariate Cox regression analyses, and results indicated that the six-gene signature was an independent prognostic factor of cancer-specific survival of patients with BCa. Functional analysis was performed based on the differentially expressed genes of low- and high-risk patients, and we found that they were enriched in lipid metabolic and cell division-related biological processes. Meanwhile, the gene set enrichment analysis (GSEA) revealed that high-risk samples were enriched in cell cycle and cancer-related pathways [G2/M checkpoint, E2F targets, mitotic spindle, mTOR signaling, spermatogenesis, epithelial–mesenchymal transition (EMT), DNA repair, PI3K/AKT/mTOR signaling, unfolded protein response (UPR), and MYC targets V2]. Lastly, we detected the relative expression of each signature in BCa cell lines by quantitative real-time PCR (qRT-PCR). As far as we know, currently, the present study is the first research that developed and validated a cancer-specific survival prognostic index based on three independent cohorts. The results revealed that this six-gene signature has a predictive ability for cancer-specific prognosis. Moreover, we also verified the relative expression of these six signatures between the bladder cell line and four BCa cell lines by qRT-PCR. Nevertheless, experiments to further explore the function of six genes are lacking.

## 1 Introduction

Human bladder cancer (BCa) is the most common urogenital system malignancy, and among the cancers related to males, it ranks fourth (Siegel et al., 2013). In China, BCa is also one of the most common urologic malignancies, and in the past few years, the incidence and mortality rates have increased gradually (Chen et al., 2015). The major risk factors for human BCa are still smoking and occupational exposures, whereas chronic infection with *Schistosoma hematobium* is relatively rare (Pang et al., 2016). BCa is divided into two types: non-muscle-invasive bladder cancer (NMIBC) and muscle-invasive bladder cancer (MIBC). Most BCa patients are diagnosed with NMIBC, which is featured as high recurrence (Prout et al., 1992). Nowadays, the common treatment for superficial BCa is transurethral resection and intravesical perfusion chemotherapy. Bacillus Calmette Guerin installation remains the gold standard of NMIBC, while appropriately 40% of patients are not sensitive to it, even 15% of patients may progress into MIBC after treating it (Seidl, 2020). What is more, the 5-year overall survival rate of patients remains at a level of 15%–20% (Cao et al., 2019). Furthermore, BCa is easy to recur and progress into MIBC. Most MIBCs were treated with radical cystectomy (Chen et al., 2015; Pang et al., 2016). As a result, the expenditure for treating BCa is huge (Sloan et al., 2020). Besides, the risks of radical cystectomy contain infection, incontinence, stones in the urethrostomy, obstruction of urine flow, damage to nearby organs, and so on (Seidl, 2020). Plenty of patients undergoing radical cystectomy generally have a poor quality of life. Therefore, it is essential to understand the critical biomarkers and key pathways governing tumor behavior for better treatment strategies and prediction of prognosis.

Due to microarray and high-throughput sequencing technology development, we could identify thousands of cancer-related genes and generate innovative insights into understanding the potential molecular mechanism of them, therefore applying them to the biomedical research field to benefit patients (Cui et al., 2015). Additionally, it is increasingly being used to search for potential biomarkers related to cancer diagnosis, treatment, and prognosis (Cancer Genome Atlas Research, 2014). In clinical practice, we found that the optional treatment strategies for patients with BCa were limited and the efficacy was not satisfactory. Hence, it is urgent to explore original target to explore new targets to provide new treatment strategies for patients with BCa. Therefore, we developed a prognostic model for BCa to predict the progression of BCa, hoping that it can provide a basis for clinical setting for BCa patients in the future.

Our study obtained mRNA expression microarray data of GSE32894 from the GEO database as the training set and another two independent test datasets, GSE13507 microarray data and The Cancer Genome Atlas (TCGA) mRNA sequencing data of BLCA. By executing univariate Cox, Least Absolute Shrinkage and Selection Operator (LASSO), and multivariate hazard Cox regression analysis, six genes related to cancer-specific survival were identified and thus constructed a six-gene prognostic index based on these genes. Another two independent test sets performed the validation of the prognostic value of the six-gene signature. Finally, we performed qRT-PCR to further verify these six genes in the bladder cell line (SV-HUV-1) and four BCa cell lines (5637, T24, UM-UC3, and J82). Our study proved that the six-gene signature could function as the independent biomarkers for the cancer-specific prognosis of human BCa and their potential roles in tumor progression.

## 2 Materials and Methods

### 2.1 Data Collection

Expressing mRNA profiles and related clinical data of human BCa were downloaded from the Gene Expression Omnibus (GEO) database (http://www.ncbi.nlm.nih.gov/geo/) (Barrett et al., 2013). Dataset GSE32894 performed on Illumina Human HT-12 V3.0 expression bead chip was used as the training set (Sjödahl et al., 2012). Dataset GSE13507 performed on Illumina human-6 v2.0 expression bead chip (Kim et al., 2010; Lee et al., 2010) and mRNA expression profiles of BLCA patients were obtained from the TCGA data portal (https://gdc-portal.nci.nih.gov/) (Ye et al., 2019) and were used as another test set. Prognostic data for all TCGA survival analyses were obtained from published papers (Liu et al., 2018).

### 2.2 Data Preprocessing

We used RMA background correction for the raw expression data for the microarray analyses at first, and log_2_ transformation and normalization were employed for processed signals. Then, we used the “affy” R package to summarize the median-polish probe sets. The Affymetrix annotation files annotated probes. For TCGA BLCA data, the gene expression data were based on the RNA-sequencing technology of IlluminaHiseq.

### 2.3 Signature Development and Validation

Firstly, we excluded samples without exact survival data. By applying the univariate hazard Cox regression analysis with survival as a dependent characteristic, the correlation between each gene expression profile and cancer-specific survival in patients was evaluated based on the training dataset (GSE32894). Here, we identified genes with *p* < 1E-6 of cancer-specific survival as prognostic gene signatures and then performed LASSO regression analysis. Genes selected from LASSO regression analysis were taken as the candidate factors, and then were subjected to perform multivariate hazard Cox regression analysis in the training dataset with cancer-specific survival as the dependent prognostic influence factor. The risk score was developed based on a linear combination of the mRNA expression level weighted by the estimated regression coefficient generated from the multivariate hazard Cox regression analysis. The formula of risk score for each patient was calculated as follows: Risk score = *β*gene1 × exprgene1 + *β*gene2 × exprgene2+ ··· + *β*geneN × exprgeneN, in which N is the number of prognostic gene signatures, expr represents the expression profiles of gene signatures, and *β* means the estimated regression coefficient of gene signatures derived from the multivariate hazard Cox regression analysis. Then, the gene signatures could calculate a risk score for each patient, and we could divide the patients into two (high- and low-risk) groups according to the median risk score. The Kaplan–Meier analysis was used to evaluate the cancer-specific survival distributions by the R “survival” package. Then, another two independent datasets were used to perform the test of the prognostic signature. GSE13507 was used to test the cancer-specific survival and TCGA BLCA data were used to test the disease-specific survival distribution. Moreover, we performed univariate Cox regression and multivariate Cox regression analysis to further verify the prognostic model’s accuracy and precision by integrating clinical features (including gender, age, tumor stage, tumor grade, and progression).

### 2.4 DEGs Analysis for High- and Low-Risk Groups

The “limma” R package was utilized to screen the distinguishingly expressed genes between high-risk and low-risk patients. The SAM (significance analysis of microarrays) with FDR (false discovery rate) < 0.05 and |log_2_ fold change (FC)| > 1 were set as the cutoff, and the DEGs were applied to further analysis.

### 2.5 Functional Analysis for DEGs

Gene ontology (GO) analysis (here, we chose the biological process) was accomplished using the R package cluster Profiler to observe the potential functions of DEGs. *p* < 0.05 was set as the cutoff criterion.

### 2.6 Gene Set Enrichment Analysis

To further analyze the potential function, the training set was performed into two groups according to the median risk score. For use with GSEA software (https://www.gsea-msigdb.org/gsea/index.jsp) (Subramanian et al., 2005), the collection of annotated gene sets of h.all.v6.1.symbol.gmat [Hallmarks] in Molecular Signatures Database (MSigDB, http://software.broadinstitute.org/gsea/msigdb/index.jsp) was chosen as the reference gene sets (Subramanian et al., 2005; Croken et al., 2014). We selected the gene sets enriched in high-risk groups or high expression level groups, and *p* < 0.05 was chosen as the cutoff criteria.

### 2.7 Gene Expression Level Evaluation

To further evaluate the gene expression level between normal bladder and BCa tissues, we used an online database GEPIA2 (http://gepia2.cancer-pku.cn/) (Tang et al., 2019). Moreover, the test set GSE13507 was used to compare the differences between normal bladder mucosae, bladder mucosae surrounding cancer, primary non-muscle invasive BCa, primary muscle invasive BCa, and recurrent non-muscle invasive tumor.

### 2.8 RNA Extraction, Reverse Transcription, and qRT-PCR

Total RNA was extracted from the nontumorous immortalized bladder cell line (SV-HUV-1) and four BCa cell lines (5637, T24, UM-UC3, and J82) using HiPure Total RNA Mini Kit (Cat. #R4111-03, Magen, China) according to the manufacturer’s instruction. The reverse transcription process was carried out with the ReverTra Ace qPCR RT Kit (Toyobo, China). The expressions of six genes were normalized to GAPDH expression. The primer sequences are listed as Supplementary Table S1.

### 2.9 Statistical Analysis

Univariate hazard Cox regression, LASSO regression, and multivariate hazard Cox regression analyses were performed to identify the prognostic factors and to establish a prognostic model. The survival curve was drawn by the Kaplan–Meier method and compared by log-rank test. ROC curve was used to evaluate the predictive power of the prognostic index. Univariate Cox regression analysis and multivariate Cox regression analysis were performed to further verify the independent prognostic value of the prognostic signature. The statistical significance of differences in qRT-PCR was compared using the Student’s *t*-test as appropriate. Bioinformatic analysis was done in the R language (version 3.6.2) and *p* < 0.05 was considered as statistically significant at two sides.

## 3 Results

### 3.1 Recognition of Prognostic Genes Related to Patients’ Cancer-Specific Survival From the Training Dataset

**FIGURE 1:**
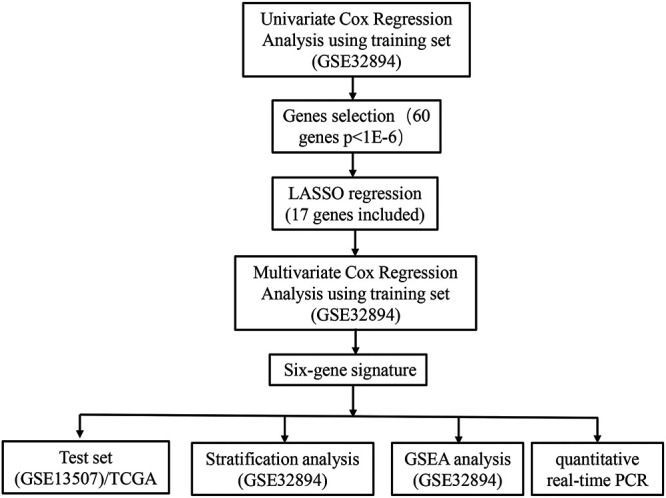
Flow chart representing the process used to select target genes included in the analysis.

**FIGURE 2:**
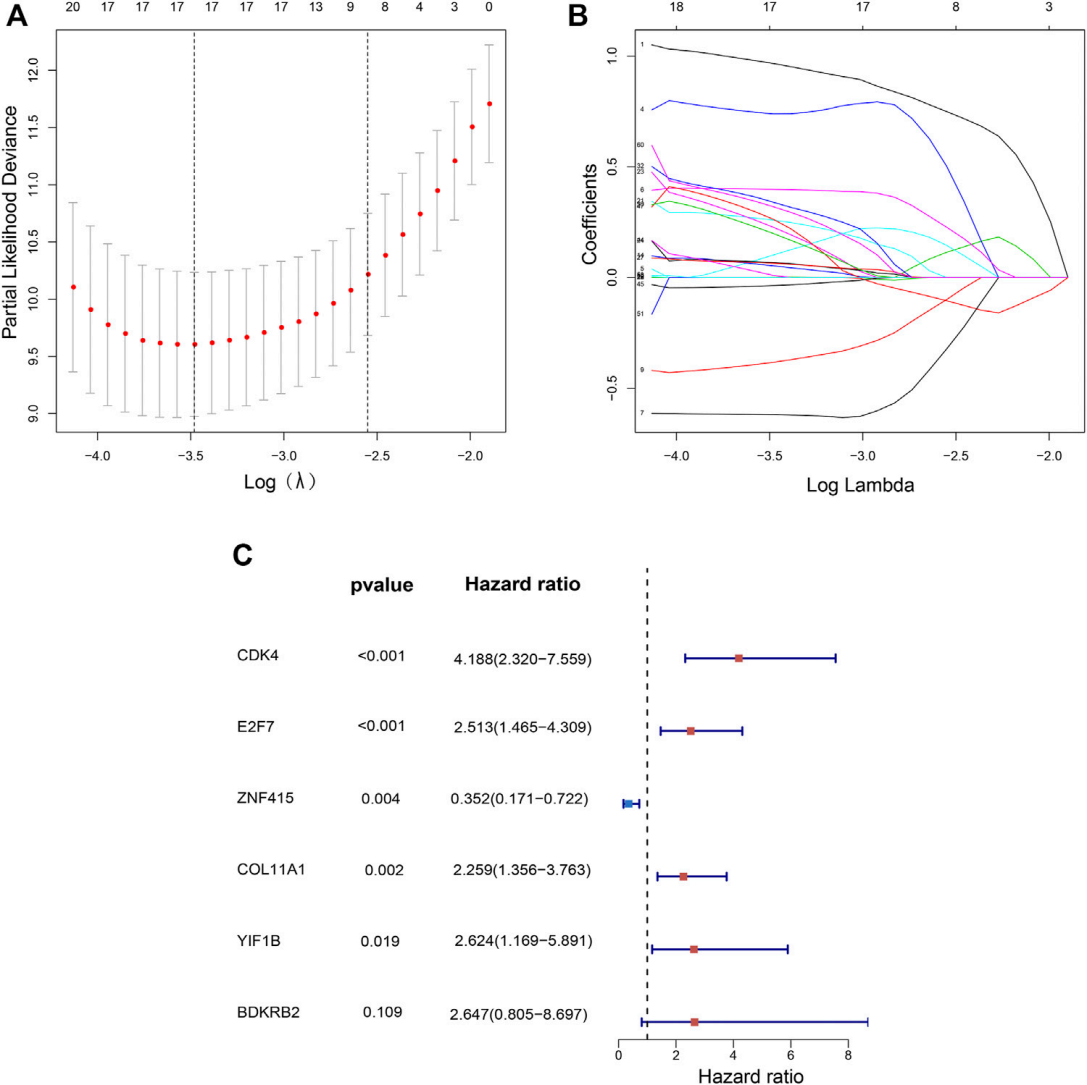
Independent prognostic-related genes selection utilizing LASSO and Multivariate cox regression. Plots of the 10-fold cross-validation error rates **(A)**. LASSO coefficient profiles of 17 prognostic-related signatures **(B)**. The multivariate hazard Cox regression analysis results show six independent prognostic-related signatures **(C)**.

The flow chart of recognition and validation of the six-gene signature is shown as Figure 1. Originally, we employed the univariate hazard Cox regression analysis to assess the connection between all gene expressions and patients’ cancer-specific survival in the training dataset (GSE32894) (Figure 1). Moreover, the result revealed that there were 60 genes significantly associated with prognosis (*p* < 1E-6), which were defined as prognostic genes. Then, the candidate genes were performed by LASSO regression (Figures 2A,B), and *CDK4*, *GUCY1A2*, *NMMT*, *E2F7*, *ZNF415*, *HTR2A*, *NUAK1*, *COL11A1*, *THOP1*, *TNFRSF6B*, *BCAT1*, *CBX2*, *CTRC*, *DHRS2*, *BDKRB2*, *YIF1B*, and *SLC22A16* were screened. Among these prognostic genes, only three genes (*ZNF415*, *HTR2A*, and *DHRS2*) with higher expression were correlated with more prolonged survival [whose *z* (coefficient) < 0], whereas other genes (*CDK4*, *GUCY1A2*, *NMMT*, *E2F7*, *NUAK1*, *COL11A1*, *THOP1*, *TNFRSF6B*, *BCAT1*, *CBX2*, *CTRC*, *BDKRB2*, *YIF1B*, and *SLC22A16*) with higher expression were lined with shorter survival [whose *z* (coefficient) > 0].

### 3.2 Establishment and Validation of a Six-Gene Signature for Predicting Patients’ Cancer-Specific Survival in the Training Dataset

**FIGURE 3:**
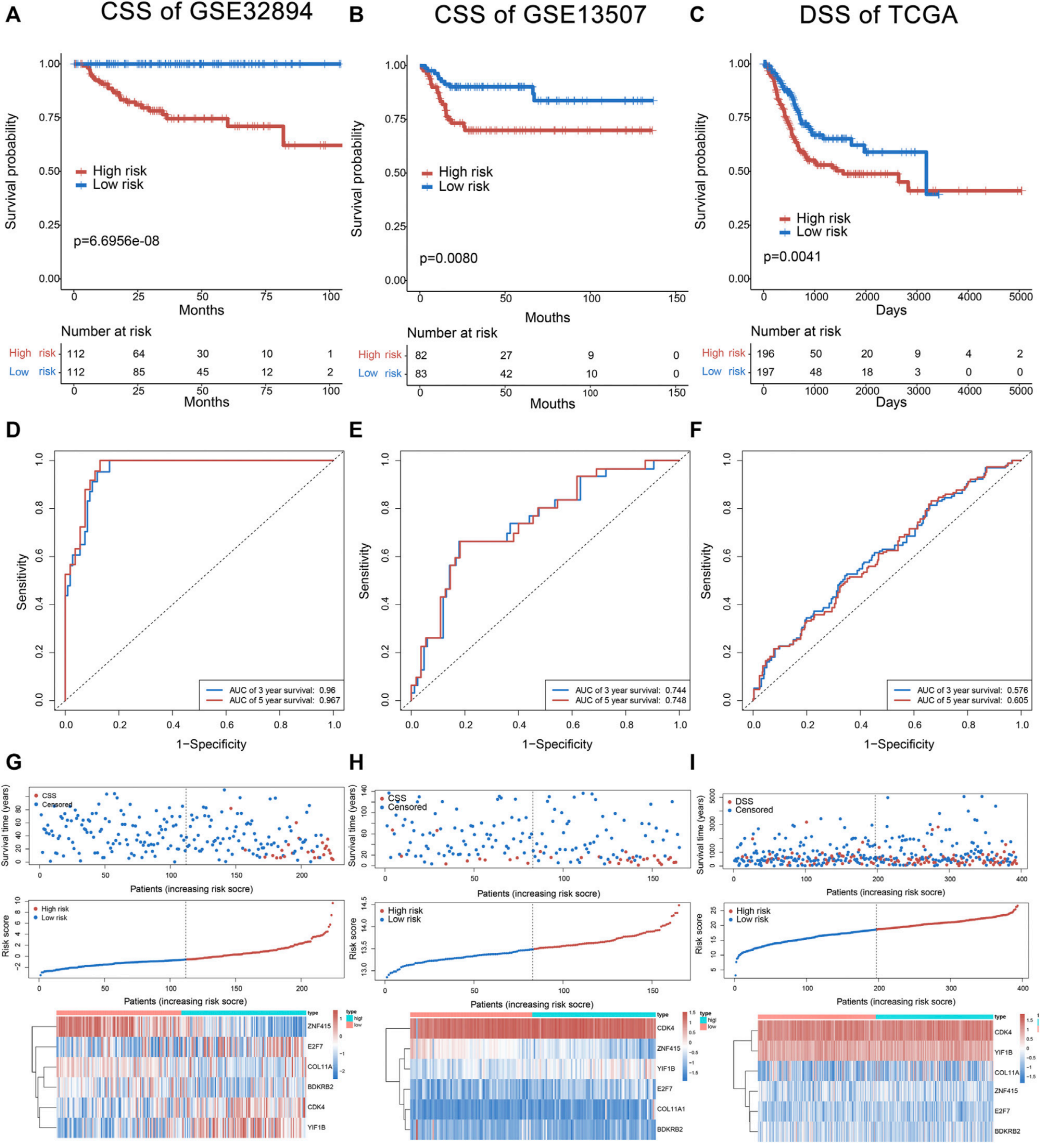
The six-gene signature in the prognosis of cancer-specific survival of bladder cancer patients in the training set and test sets (GSE13507 and TCGA). The Kaplan–Meier curves of survival between high-risk and low-risk patients in the training set and test sets **(A–C)**. The ROC curve for survival prediction by the six-gene signature within 3 and 5 years as the defining point in the training set and test sets **(D–F)**. The six-gene risk score distribution, survival of patients, and heatmap of the six-gene expression profiles in the training set and test sets **(G–I)**.

Multivariate hazard Cox regression analysis was further used to analyze those 17 prognostic genes and then selected genes independently related to cancer-specific survival. Eventually, we screened six genes (*CDK4*, *E2F7*, *COL11A1*, *BDKRB2*, *YIF1B*, and *ZNF415*) as the independent factor and established a prognostic model (Figure 2C). *Via* integrating the expression of those six genes and the estimated regression coefficient, we then obtained the following calculation model: Risk score = (1.43215589574675 × expression of *CDK4*) + (0.921330280956022 × expression of *E2F7*) + (−1.04548254381182 × expression of *ZNF415*) + (0.814780461026126 × expression of *COL11A1*) + (0.973314699914422 × expression value of *BDKRB2*) + (0.964685342668668 × expression value of *YIF1B*). With the six-gene signature, the risk score for each patient with BCa in the training dataset could be calculated and ranked from the largest to the smallest. Based on the median risk score (0.630561), 224 BCa patients in the training dataset were divided into a high-risk group (*n* = 112) and a low-risk group (*n* = 112). There was an obvious difference (*p* = 6.6956E-08) in patients’ cancer-specific survival between the high-risk and the low-risk groups (Figure 3A). Moreover, we could observe that those ranked into the high-risk group had remarkably shorter survival (median 28.84 months) than those in the low-risk group (median 44.28 months). The time-dependent ROC curve was carried out for 3- and 5-year cancer-specific survival to evaluate the efficacy of the six-gene signature for predicting the cancer-specific survival. The AUCs for the six-gene signature at the cancer-specific survival of 3 and 5 years were 0.96 and 0.967, respectively (Figure 3D). The distribution of the risk score, cancer-specific survival time, and six genes’ expression profiles in the training dataset are shown in Figure 3G, ranked with the increasing risk score. We could find that high-risk patients lived shorter than low-risk patients, and meanwhile, the expression level of patients had a similar trend in five genes (*CDK4*, *E2F7*, *COL11A1*, *BDKRB2*, and *YIF1B*), elevating with the increasing risk score, while *ZNF415* demonstrated the opposite trend.

### 3.3 Validation of the Six-Gene Signature in the Test Sets

Cancer-specific survival of GSE13507 was utilized to test and verify the prognostic efficacy of the six-gene signature for cancer-specific survival prediction; 165 patients of the test set (GSE13507) were classified into the high-risk group (*n* = 83) and low-risk group (*n* = 82) according to the same formula generating from GSE32894. The result showed a significant difference (*p* = 0.0080; median 29.37 vs. 46.835 months) in cancer-specific survival between high-risk and low-risk groups (Figure 3B). The AUC for the six-gene signature was 0.744 and 0.748 at the cancer-specific survival of 3 and 5 years, respectively, in the test set (GSE13507) (Figure 3E). The distribution of the risk score, cancer-specific survival time, and six genes’ expression profiles in the test set of GSE13507 are shown in Figure 3H, ranked with the increasing risk score. In addition, the disease-specific survival of TCGA was used to verify the accuracy of the six-gene signature. As shown in Figure 3C, patients in high risk had a lower survival rate than those in low risk (*p* = 0.0041). The AUC for the six-gene signature was 0.576 and 0.606 at the disease-specific survival of 3 and 5 years, respectively (Figure 3F). The distribution of the risk score, disease-specific survival time, and six genes’ expression profiles in TCGA are shown in Figure 3I. Above all, the results indicated the good reliability and reproducibility of the six-gene prognostic model for forecasting cancer-specific survival for patients with BCa.

### 3.4 Independent Prognostic Analysis of Prognostic Signature

**FIGURE 4:**
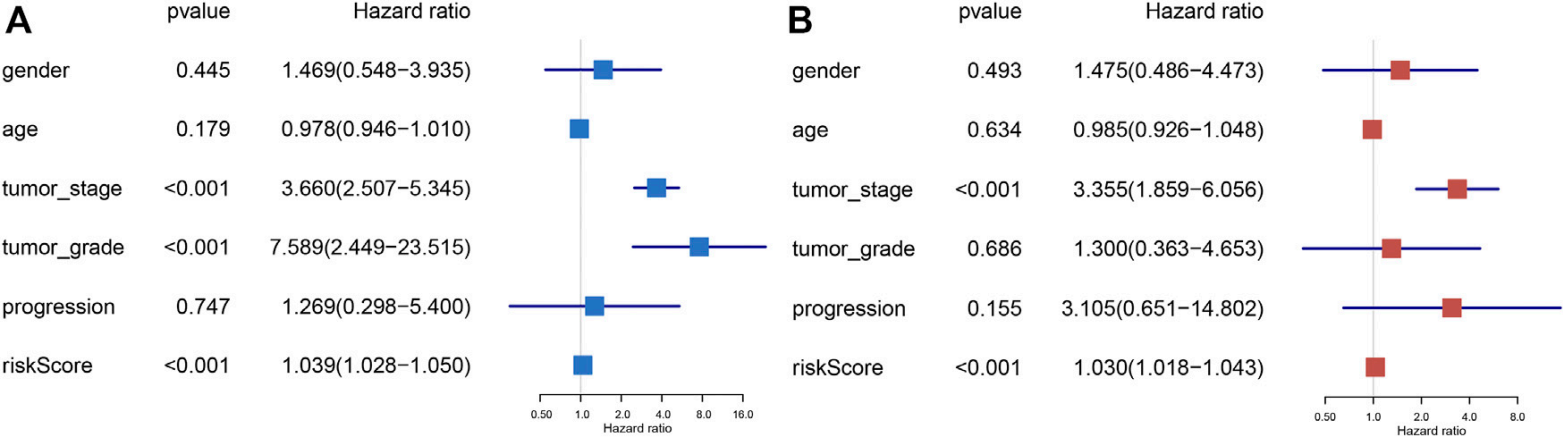
Univariate Cox and multivariate Cox regression of the prognostic signature integrating with clinical parameters, including gender, age, tumor stage, tumor grade, and progression. Univariate Cox regression analysis for signature and clinical variants **(A)**. Multivariate Cox regression analysis for signature and clinical features **(B)**.

In order to explore whether the prognostic index is an independent prognostic factor, we conducted univariate Cox regression analysis and multivariate Cox regression analysis by integrating several clinicopathological characteristics, including gender, age, tumor stage, tumor grade, and progression. The results indicated that prognostic signature was significantly associated with the cancer-specific survival of BLCA not only in univariate Cox regression analysis (*p* < 0.001) (Figure 4A), but also in multivariate Cox regression analysis (*p* < 0.001) (Figure 4B). In summary, the six-gene prognostic model can be seen as an independent prognostic indicator of BLCA.

### 3.5 Clinicopathological Correlation Analysis of Prognostic Signature

**FIGURE 5:**
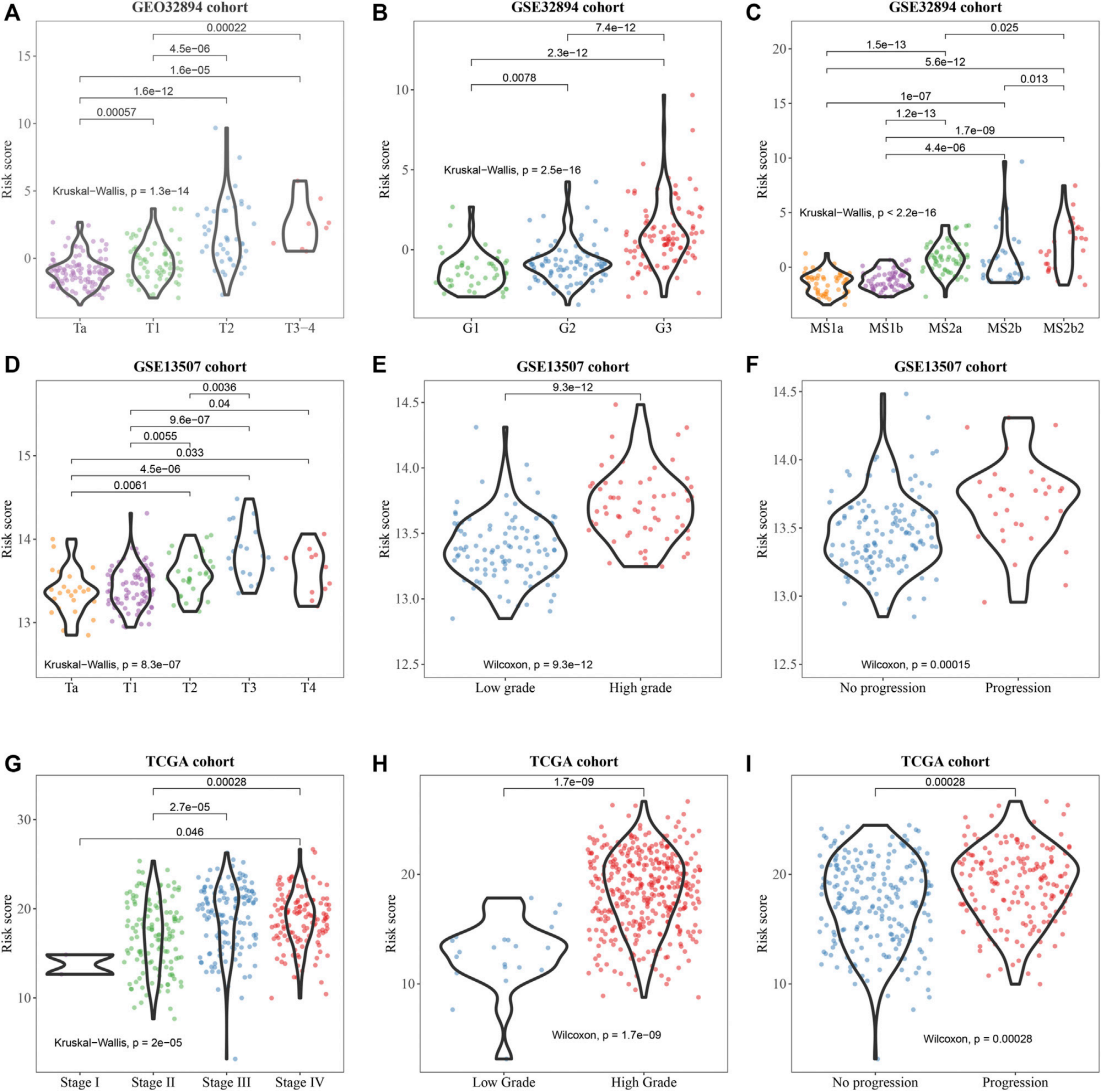
Clinicopathological significance of the prognostic signature of bladder cancer in the training set (GSE32894) and test sets (GSE13507 and TCGA). *p* values were statistically significant at T-stage **(A, D)**, grade **(B, E, H)**, molecular subty*p*e **(C)**, pathological stage **(G)**, and progression **(F, I)**.

Subsequently, the correlation of the six-gene signature with clinicopathological features and its prognostic significance were analyzed in the training set and two test sets. We observed that the signature was significantly correlated with BCa divided by T-stage in GSE32894 and GSE13507 (Figures 5A,D) grade in all sets (Figures 5B,E,H). In addition, we found that it was also associated with molecular subtype in GSE32894 (Figure 5C), pathological stage in TCGA (Figure 5G) and progression in test sets GSE13507 (Figure 5F) and TCGA (Figure 5I).

### 3.6 Stratified Analyses of the Six-Gene Signature for Cancer-Specific Survival Prediction of Other Clinical Characteristics

**FIGURE 6:**
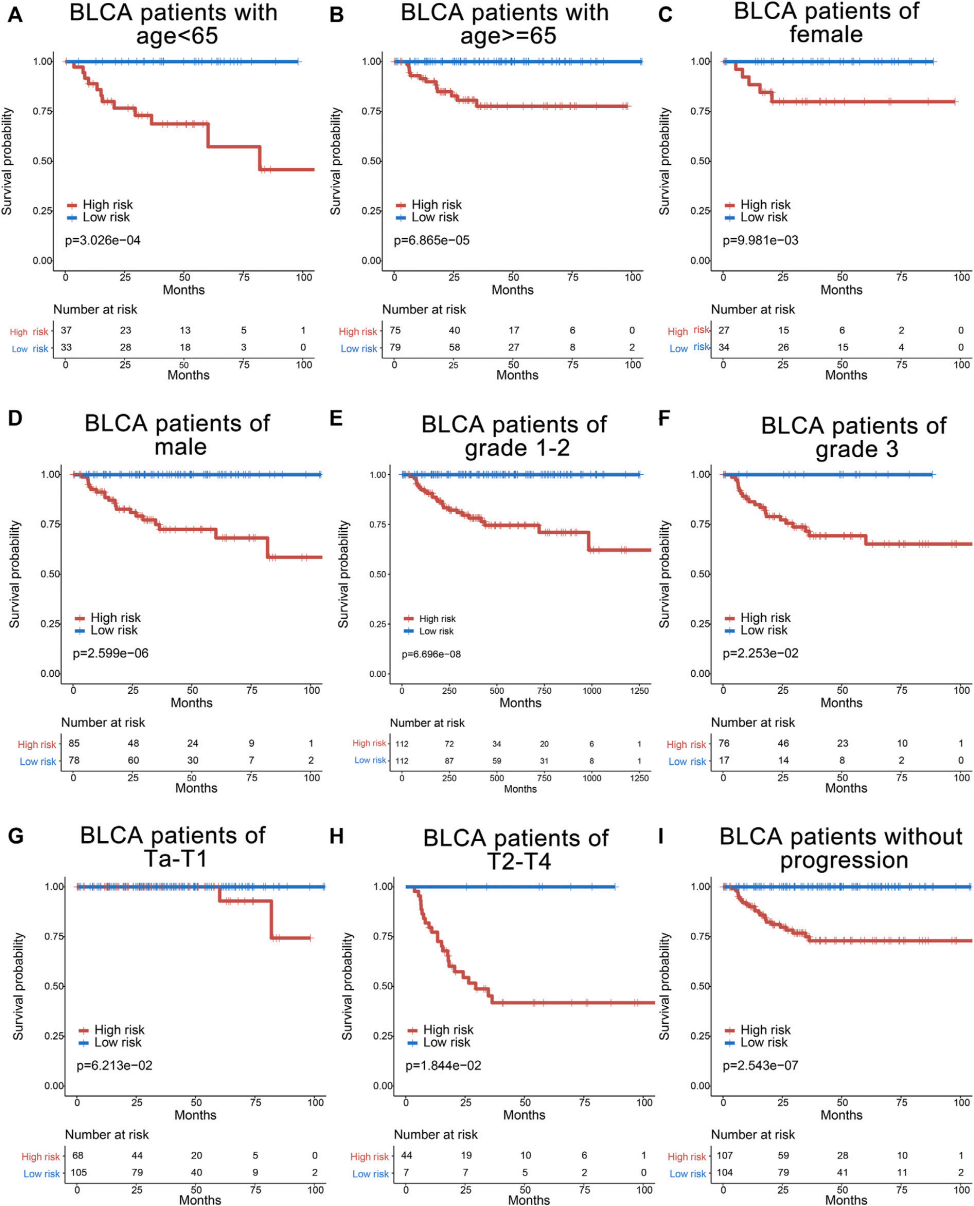
Survival analyses of bladder cancer patients stratified by age, gender, grade, T stage, and tumor progression with the six-gene signature in GSE32894. The Kaplan–Meier curves for the young (age <65) and old (age ≥65) groups **(A,B),** for the female and male patients **(C,D)**, for the grade 1–2 and grade 3 groups **(E,F)**, for the low stage (Ta and T1) and high stage (T2–T4) groups **(G,H)**, and for the non-progression (without progression to higher stage or grade) group **(I)**.

Furthermore, to assess the prognostic value of the six-gene index, the stratified analyses were performed by using clinical information including age, gender, tumor grade, tumor stage, node status, and tumor progression. All 224 BCa patients were firstly stratified by age into the younger dataset (<65 years old, *n* = 70) and the elder dataset (≥65 years old, *n* = 154), by gender into a female dataset (*n* = 61) and male dataset (*n* = 163), and by tumor grade into grade 1–2 (*n* = 129) and grade 3 (*n* = 93). The prognostic power of the six-gene signature was significant in the younger dataset, the elder dataset, the female dataset, the male dataset, the grade 1–2 dataset, and the grade 3 dataset (Figures 6A–F). Based on the tumor stage, patients were categorized into low stage (Ta and T1, *n* = 173) and high stage (T2–T4, *n* = 51). Meanwhile, patients were also stratified by node status into N0 (*n* = 26) and N+ (*n* = 20) and by tumor progression status into non-tumor progression dataset (*n* = 211) and tumor progression dataset (*n* = 13). Interestingly, a similar significant prognostic value could be observed in the high-stage dataset and patients without progression dataset (Figures 6H,I). Otherwise, the prognosis of the low-stage dataset, and N0 and N+ and tumor progression datasets had no significance (data not shown), which may be due to the limited patients.

### 3.7 DEGs for High- and Low-Risk Patients

**FIGURE 7:**
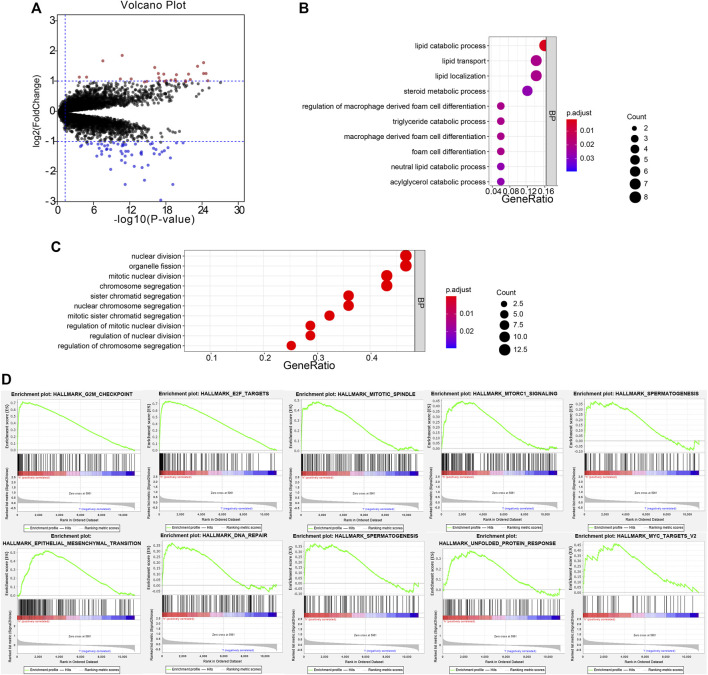
Functional annotation of DEGs. The volcano plot based on the differentially expressed genes **(A)**. Biological process analysis of the remarkable association of down- and upregulated genes **(B,C)**. The top 10 enriched pathways in the high-risk group were analyzed by gene set enrichment analysis **(D)**.

To investigate the potential function of the six prognostic genes, samples in the training set GSE32894 were divided into two groups according to the risk score. Under the threshold of FDR < 0.05 and |log_2_ FC| > 1, a total of 82 DEGs were screened (54 downregulated and 28 upregulated). The volcano plot presented the differential expressed signatures between high- and low-risk groups (Figure 7A).

### 3.8 Functional Annotation of the DEGs

The biological process of down- and upregulated genes between high- and low-risk groups is visualized in Figures 6B,C, respectively. In the low-risk group, the biological process was enriched in lipid catabolic process, lipid transport, lipid localization, steroid metabolic process, regulation of macrophage-derived foam cell differentiation, triglyceride catabolic process, macrophage-derived foam cell differentiation, foam cell differentiation, neutral lipid catabolic process, and acylglycerol catabolic process. In the high-risk group, the biological processes were significantly enriched in the nuclear division, organelle fission, mitotic nuclear division, chromosome segregation, sister chromatid segregation, nuclear chromosome segregation, mitotic sister chromatid segregation, regulation of mitotic nuclear division, regulation of nuclear division, and regulation of chromosome segregation. Moreover, GSEA analysis was performed, and it revealed that high-risk samples were enriched in G2/M checkpoint, E2F targets, mitotic spindle, mTOR signaling, spermatogenesis, EMT, DNA repair, PI3K/AKT/mTOR signaling, UPR, and MYC targets V2 (Figure 7D).

### 3.9 Relative Expression of Six Genes in Bladder Cell Line and for BCa Cell Lines

**FIGURE 8:**
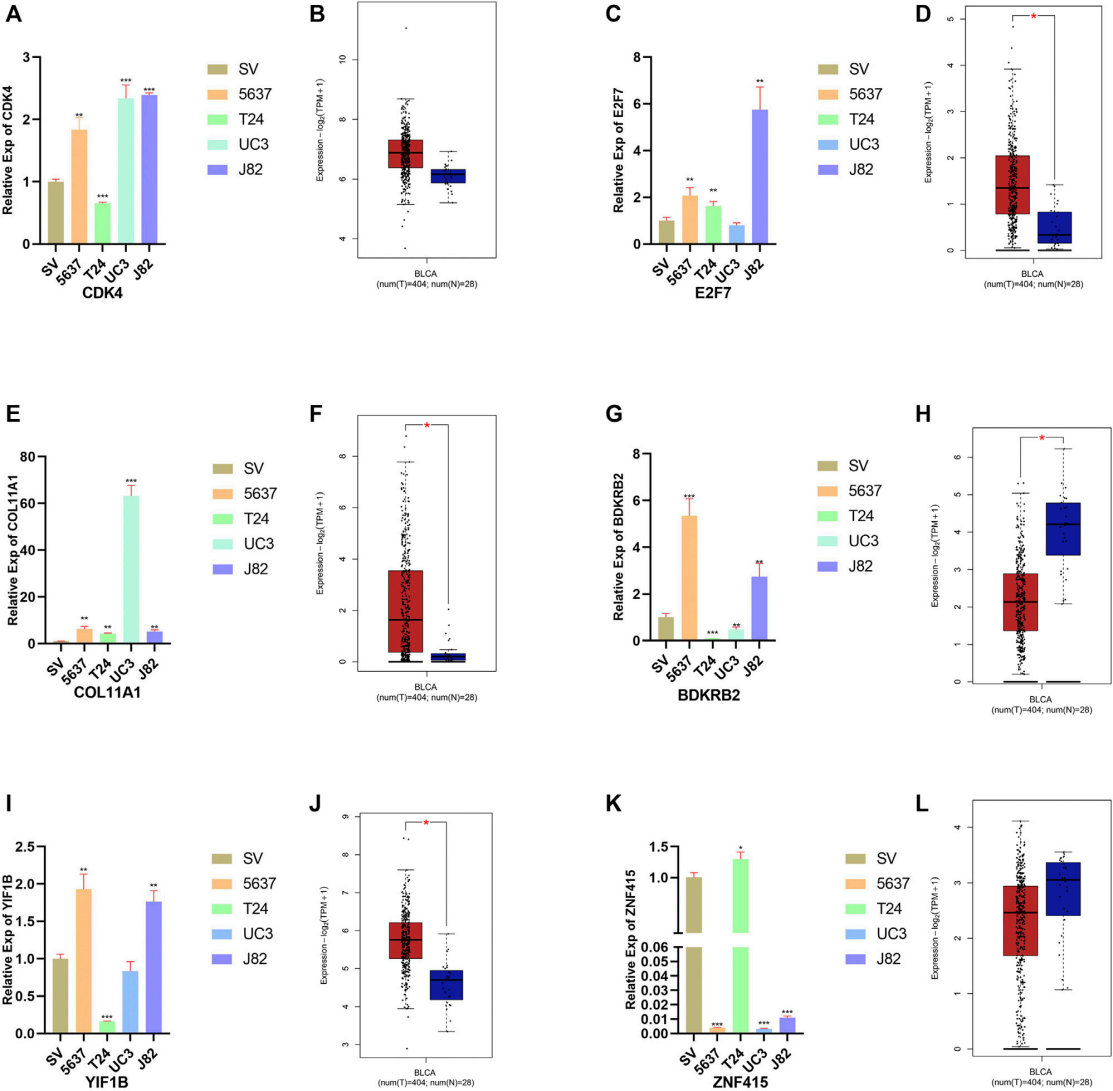
Relative expression of six signatures in the bladder cell line and four BCa cell lines. Expression of *CDK4*, *E2F7*, *COL11A1*, *BDKRB2*, *YIF1B*, and *ZNF415* in the bladder cell line (SV-HUV-1 (SV in short)) and four BCa cell lines (5637, T24, UM-UC3 (UC3 in short), and J82) **(A, C, E, G, I, K)**. Relative expression of *CDK4*, *E2F7*, *COL11A1*, *BDKRB2*, *YIF1B*, and *ZNF415* in normal bladder and BCa cancer tissues in GEPIA2 **(B, D, F, H, J, L)**.

The results of qRT-PCR and expression profiles between the normal bladder and BCa tissues of six signatures are shown in Figure 7. Compared with normal bladder epithelial cell line (SV-HUV-1), the level of *CDK4*, *E2F7*, *COL11A1*, *BDKRB2*, and *YIF1B* was upregulated in most BCa cell lines (Figures 8A,C,E,G,I). On the contrary, the level of *ZNF415* (Figure 7K) was downregulated, compared with SV-HUV-1, which were in line with our above contents. In GEPIA2, the expression of *CDK4*, *E2F7*, *COL11A1*, and *YIF1B* was upregulated in BCa tissues compared with normal bladder tissues (Figures 8B,D,F,J), while the level of *BDKRB2* and *ZNF415* showed an opposite outcome (Figures 7H,L). FDR < 0.05 and |log_2_ FC| > 1 were used as thresholds for judging the significance of gene expression differences in GEPIA2. The results of qRT-PCR were roughly in line with the consequences of GEPIA2 and our above contents that higher expression was related with shorter survival, such as *CDK4* and *E2F7*, and that higher expression was connected with longer survival, for instance, *ZNF415*.

## 4 Discussion

With the development of molecular biomarkers, like tumoral suppressors or oncogenes, which are less expensive and less invasive, we could detect human BCa or predict patients’ outcomes more easily. Additionally, together with the currently used cystoscopy, patients could be provided a better chance for appropriate therapies.

We identified six genes (*CDK4*, *E2F7*, *COL11A1*, *BDKRB2*, *YIF1B*, and *ZNF415*) that were significantly associated with BCa prognosis and developed a six-gene signature. Based on the six-gene signature, we observed that patients in the high-risk group had shorter cancer-specific survival than the low-risk group. Furthermore, the high-risk group also showed worse cancer-specific survival than the low-risk group in patients with other clinical features (age, gender, tumor grade, tumor stage and node status, and tumor progression). In addition, the results of univariate Cox regression and multivariate Cox regression analysis showed that the six-gene prognostic signature was an independent prognostic factor of BLCA.

All of the six genes have vital functions. *CDK4*, a Ser/Thr protein kinase family member and its partner CDK6, is a key player in cell cycle progression (Sheppard and McArthur, 2013). It is reported that CDKs could induce genomic and chromosomal instability and unscheduled proliferation, which attach great importance to oncogenesis (Malumbres and Barbacid, 2009). *E2F7*, a member of the E2F family, plays an essential role in regulating the cell cycle (Chen et al., 2009). It is also reported that *E2F7* is a unique repressor of a subset of E2F target genes whose products are required for cell cycle progression (Di Stefano et al., 2003). Mitxelena et al. reported that *E2F7* controlled a new regulatory network involving transcriptional and post-transcriptional mechanisms to restrain cell cycle progression through repression of proliferation-promoting miRNAs (Mitxelena et al., 2016). Chu et al. demonstrated that upregulated *E2F7* restrains the level of miR-15a/16 and therefore promotes Cyclin E1 and Bcl-2, thereby bringing out tamoxifen resistance. *COL11A1* is a part of type XI collagen, which acts as a vital role in skeletal development. Other studies have shown that high expression of *COL11A1* is related to poor clinical prognosis in diverse cancers. Overexpression of *COL11A1* could accelerate cancer cell proliferation, invasion, migration, and metastasis, and resist chemotherapy sensitivity (Cheon et al., 2014; Wu et al., 2014; Wu et al., 2019; Wang et al., 2020; Nallanthighal et al., 2021). *BDKRB2*, an angiogenesis-related gene, demonstrated as a direct IRX1 target gene and was reported to be involved in gastric cancer progression (Jiang et al., 2011). A previous study revealed that bradykinin could upregulate the levels of TRPM7 and MMP2 to promote the invasion and migration of hepatocellular carcinoma cells (Chen et al., 2016). *YIF1B* is a gene related to nervous development, whose mutation could lead to neurodevelopmental syndrome (Diaz et al., 2020). With the development of bioinformatics, *YIF1B* was gradually exploited to predict clinical prognosis for cancer patients (Liu et al., 2020; Jia et al., 2021). *ZNF415*, a member of zinc finger proteins, was reported to play an essential role in AP-1 and p53-mediated transcriptional activity regulation (Cheng et al., 2006). In addition, Omura et al. observed that *ZNF415*, as a methylated promoter, is involved in pancreatic adenocarcinoma (Omura et al., 2008).

In the test set, we could observe that five (*CDK4*, *E2F7*, *BDKRB2*, *YIF1B*, and *ZNF415*) of these six signatures were differentially expressed between BCa tissues and normal bladder tissues. Moreover, *CDK4* and *YIF1B* were discovered as the biomarkers to distinguish the recurrent BCa and BCa.

To further study the potential function, GO analysis and GSEA were performed. GO biological process enrichment analysis for differentially expressed genes between high- and low-risk groups indicated that the lipid metabolic process and associated terms were enriched in the low-risk group, whereas cell division and interrelated terms were enriched in the high-risk group. Cell division is essential for tumor development and progression. Many times, cell divisions were asymmetric, containing protein content, cell size, or developmental potential, leading to cancer incidence and other diseases (Chia et al., 2008; Neumuller and Knoblich, 2009). Because DNA is the only cellular component that can accumulate and transmit changes throughout life (from zygote to death), it was soon accepted that carcinogenesis of cancer requires a multi-step accumulation of DNA (López-Lázaro, 2018). Conferring to the GSEA analysis, we found that the G2/M checkpoint, E2F targets, and mitotic spindle, which regulated the cell cycle, were enriched. Meanwhile, other functional pathways were enriched either. mTOR signaling activated protein synthesis by phosphorylating 4E-BP1 and S6K1 (Holz, 2012); regulated metabolic pathways on transcriptional, translational, and posttranslational levels (Peng et al., 2002); promoted lipid and cholesterol synthesis (Porstmann et al., 2008); and was involved in autophagy (Codogno and Meijer, 2005), which was essential for the cancer progression. EMT signaling pathway was closely related to the progress of cancer, which promoted the mobility, invasion, and resistance to apoptotic stimuli to accelerate the metastasis of cancer cells (Mittal, 2018; Lu and Kang, 2019). DNA repair was crucial to maintain the survival and growth of cells. Lack of DNA repair pathway led to the change of genome, which favored cancer cell proliferation (Klinakis et al., 2020). The PI3K/AKT/mTOR signaling pathway was implicated in a wide spectrum of cancers, neurological diseases, and proliferative disorders (Alayev and Holz, 2013). The PI3K/AKT/mTOR pathway regulated cell proliferation, growth, cell size, metabolism, and motility (Alzahrani, 2019). UPR was the potential driver for cancer and other chronic metabolic diseases. UPR delivered the information of protein folding status to the nucleus and cytosol to induce cell apoptosis when the body is in a state of chronic injury and consumption (Hetz et al., 2020). MYC was demonstrated to promote cell proliferation. High targets V2 was able to act as an indicator to predict the clinical prognosis (Schulze et al., 2020).

The six-gene prognostic model can effectively predict the prognosis of patients with BCa and may provide a clinical setting for individualized treatment of BCa in the future. Moreover, we verified the relative expression of these six signatures between the bladder cell line and four BCa cell lines by qRT-PCR. However, we have to admit that our research is insufficient. First of all, we only have TCGA and one GEO dataset to validate the prognostic index, and we have not further validated our model through other databases such as ICGC and Oncomine. In addition, the cell function experiments of the six genes in BCa have not been explored in depth.

## 5 Conclusion

In conclusion, those six genes are able to distinguish human BCa tissues and normal tissues, and their expression signature combination could also possess a predictive ability for the cancer-specific prognosis.

## Data Availability

The datasets presented in this study can be found in online repositories. The names of the repository/repositories and accession number(s) can be found in the article/Supplementary Material.

## Abbreviations

BCa, Bladder cancer; CSS, Cancer-specific survival; DEG, Differentially expressed gene; DSS, Disease-specific survival; FC, Fold change; FDR, False discovery rate; GEO, Gene Expression Omnibus; GEPIA, Gene Expression Profiling Interactive Analysis; GSEA, Gene Set Enrichment Analysis; ICGC, International Cancer Genome Consortium; qRT-PCR, Quantitative real-time PCRRMA, Robust Multi-array Average; ROC curve, Receiver operating characteristic curve; SAM, Significance Analysis of Microarrays; TCGA, The Cancer Genome Atlas.
