# Supplementary material for: Development and Validation of a Six-Gene Prognostic Signature for Bladder Cancer
Source: Front Genet. 2021 Dec 6;12:758612. doi: 10.3389/fgene.2021.758612 (PMC8685517; doi:10.3389/fgene.2021.758612)
Supplement: Supplementary file 1 [file DataSheet2.ZIP › raw data/Code/code.docx]

##########Establishment of six-gene signature model ##################

setwd("D:/")

**1.**#######Univariate#######

library(survival)

pFilter=0.05

rt=read.table("GSE32894expTime.txt",header=T,sep="\t",check.names=F,row.names=1,encoding="UTF-8")

print("success read table")

outTab=data.frame()

sigGenes=c("futime","fustat")

for(i in colnames(rt[,3:ncol(rt)])){

print(i)

if(i != "RP11-45B20.2" && i != "bA16L21.2.1" && i != "dJ341D10.1")

{

i = strsplit(i,split = ".1",fixed = T)

i = toString(i)

i = strsplit(i,split = ".2",fixed = T)

i = toString(i)

i = strsplit(i,split = ".3",fixed = T)

i = toString(i)

i = strsplit(i,split = ".4",fixed = T)

i = toString(i)

i = strsplit(i,split = ".5",fixed = T)

i = toString(i)

i = strsplit(i,split = ".6",fixed = T)

i = toString(i)

i = strsplit(i,split = ".7",fixed = T)

i = toString(i)

i = strsplit(i,split = ".8",fixed = T)

i = toString(i)

i = strsplit(i,split = ".9",fixed = T)

i = toString(i)

}

cox <- coxph(Surv(futime, fustat) ~ rt[,i], data = rt)

coxSummary = summary(cox)

coxP=coxSummary$coefficients[,"Pr(>|z|)"]

if(coxP<pFilter){

sigGenes=c(sigGenes,i)

outTab=rbind(outTab,

cbind(id=i,

HR=coxSummary$conf.int[,"exp(coef)"],

HR.95L=coxSummary$conf.int[,"lower .95"],

HR.95H=coxSummary$conf.int[,"upper .95"],

pvalue=coxSummary$coefficients[,"Pr(>|z|)"])

)

}

}

write.table(outTab,file="UniCox.txt",sep="\t",row.names=F,quote=F)

uniSigExp=rt[,sigGenes]

uniSigExp=cbind(id=row.names(uniSigExp),uniSigExp)

write.table(uniSigExp,file="UniSigExp.txt",sep="\t",row.names=F,quote=F)

**2**.########LASSO regression **(Figure 2A-B)**#######

rt=read.table("UniSigExp.txt",header=T,sep="\t",row.names=1)

gene=read.table("gene p less E-6.txt",header=F)

rt=rt[,c("futime","fustat",as.vector(gene[,1]))]

x=as.matrix(rt[,c(3:ncol(rt))])

y=data.matrix(Surv(rt$futime,rt$fustat))

fit <- glmnet(x, y, family = "cox", maxit = 1000)

pdf("Figure 2A lambda.pdf")

plot(fit, xvar = "lambda", label = TRUE)

dev.off()

cvfit <- cv.glmnet(x, y, family="cox", maxit = 1000)

pdf("Figure 2B cvfit.pdf")

plot(cvfit)

abline(v=log(c(cvfit$lambda.min,cvfit$lambda.1se)),lty="dashed")

dev.off()

coef <- coef(fit, s = cvfit$lambda.min)

index <- which(coef != 0)

actCoef <- coef[index]

lassoGene=row.names(coef)[index]

geneCoef=cbind(Gene=lassoGene,Coef=actCoef)

write.table(geneCoef,file="geneCoef.txt",sep="\t",quote=F,row.names=F)

**3.**#######Multivariate **(Figure 2C)** #######

rt=read.table("pre-multiCox.txt",header=T,sep="\t",check.names=F,row.names=1)

multiCox=coxph(Surv(futime, fustat) ~ ., data = rt)

multiCox=step(multiCox,direction = "both")

multiCoxSum=summary(multiCox)

outTab=data.frame()

outTab=cbind(

coef=multiCoxSum$coefficients[,"coef"],

HR=multiCoxSum$conf.int[,"exp(coef)"],

HR.95L=multiCoxSum$conf.int[,"lower .95"],

HR.95H=multiCoxSum$conf.int[,"upper .95"],

pvalue=multiCoxSum$coefficients[,"Pr(>|z|)"])

outTab=cbind(id=row.names(outTab),outTab)

outTab=gsub("`","",outTab)

write.table(outTab,file="multiCox.txt",sep="\t",row.names=F,quote=F)

rt <- read.table("multiCox.txt",header=T,sep="\t",row.names=1,check.names=F)

gene <- rownames(rt)

hr <- sprintf("%.3f",rt$"HR")

hrLow <- sprintf("%.3f",rt$"HR.95L")

hrHigh <- sprintf("%.3f",rt$"HR.95H")

Hazard.ratio <- paste0(hr,"(",hrLow,"-",hrHigh,")")

pVal <- ifelse(rt$pvalue<0.001, "<0.001", sprintf("%.3f", rt$pvalue))

pdf(file=" Figure 2C forest.pdf", width = 6,height = 4.5)

n <- nrow(rt)

nRow <- n+1

ylim <- c(1,nRow)

layout(matrix(c(1,2),nc=2),width=c(3,2))

xlim = c(0,3)

par(mar=c(4,2.5,2,1))

plot(1,xlim=xlim,ylim=ylim,type="n",axes=F,xlab="",ylab="")

text.cex=0.8

text(0,n:1,gene,adj=0,cex=text.cex)

text(1.5-0.5*0.2,n:1,pVal,adj=1,cex=text.cex);text(1.5-0.5*0.2,n+1,'pvalue',cex=text.cex,font=2,adj=1)

text(3,n:1,Hazard.ratio,adj=1,cex=text.cex);text(3,n+1,'Hazard ratio',cex=text.cex,font=2,adj=1,)

par(mar=c(4,1,2,1),mgp=c(2,0.5,0))

xlim = c(0,max(as.numeric(hrLow),as.numeric(hrHigh)))

plot(1,xlim=xlim,ylim=ylim,type="n",axes=F,ylab="",xaxs="i",xlab="Hazard ratio")

arrows(as.numeric(hrLow),n:1,as.numeric(hrHigh),n:1,angle=90,code=3,length=0.05,col="darkblue",lwd=2.5)

abline(v=1,col="black",lty=2,lwd=2)

boxcolor = ifelse(as.numeric(hr) > 1, '#C6524A', '#2874C5')

points(as.numeric(hr), n:1, pch = 15, col = boxcolor, cex=1.3)

axis(1)

dev.off()

########GSE32894 Plots ###############################################

####Survival curve **(Figure 3A)** ###

library(dplyr)

library(survminer)

library(survival)

GSE32894<-read.delim(file = "D:/GSE32894.txt",header = T,row.names = 1)

GSE32894clinicaldata<-read.delim(file = "D:/GSE32894clinical.txt",header = T,row.names = 1)

model<-read.delim("D:/multiCox.txt",header = 1)

rownames(model)<-model$id

model<-model %>% dplyr::select("coef")

model<-as.matrix(model)

GSE32894<-GSE32894[,rownames(GSE32894clinicaldata)]

data<-t(GSE32894)

GSE32894data<-GSE32894clinicaldata

nall<-nrow(data)

RT<-data

classifier<-matrix(data=0,ncol=nall,nrow=(dim(model)[1]+1))

t<-t(RT)

colnames(classifier)<-colnames(t)

intersect(rownames(model),rownames(GSE32894))

for(j in 1:nall)

for(i in 1:dim(model)[1])

{classifier[i+1,j]=classifier[i,j]+model[i]*t[as.matrix(rownames(model))[i],j]}

classifier[dim(model)[1]+1,]

allscore<-classifier[dim(model)[1]+1,]

GSE32894data$score<-allscore

GSE32894data$DOD<-as.factor(GSE32894data$DOD)

GSE32894data$NEW_group=ifelse(GSE32894data$score > median(GSE32894data$score),'high risk','low risk')

table(GSE32894data$NEW_group)

fit <- survfit(Surv(GSE32894data$DOD.time, GSE32894data$DOD=="1")~GSE32894data$NEW_group, data=GSE32894data)

fit

my.surv <-Surv(GSE32894data$DOD.time, GSE32894data$DOD=="1")

data.survdiff <- survdiff(my.surv~GSE32894data$NEW_group,data=GSE32894data)

p.val = 1 - pchisq(data.survdiff$chisq,length(data.survdiff$n) - 1)

p.val

pdf("GSE32894_Figure3A.pdf",width=6.23,height=6.57,file = "D:/GSE32894_Figure3A.pdf")

ggsurvplot(fit, size=2.2,

legend.labs = c("High risk","Low risk"),

pval=paste("p=",round(p.val,4)),

conf.int=F,risk.table=T,risk.table.col="black" ,

pval.size=5,pval.coord= c(-0.1,0.1),

legend=c(0.2,0.3),

legend.title="",

font.legend=13,xlab="Months",ggtheme = theme_survminer(),

test.for.trend = T,linetype = 1,cumevents = F,censor.shape=3,

censor.size=3,

pval.method.size=10,pval.method=T,pval.method.coord= c(0,0.15),

risk.table.fontsize=4, font.x=18, font.y=18,

tables.y.text=T, palette = c("#C6524A","#2874C5"))

dev.off()

####Survival Riskplots **(Figure 3G)** ####

library(pheatmap)

rt=GSE32894data[order(GSE32894data$score),]

###Risk Curve

riskClass=rt[,"NEW_group"]

lowLength=length(riskClass[riskClass=="low risk"])

highLength=length(riskClass[riskClass=="high risk"])

line=rt[,"score"]

pdf(file="GSE32894_riskScore_Figure3G.pdf",width = 10,height = 4)

plot(line,

type="p",

pch=20,

xlab="Patients (increasing risk socre)",

ylab="Risk score",

col=c(rep("#2874C5",lowLength),

rep("#C6524A",highLength)))

abline(h=median(rt$riskScore),v=lowLength,lty=2)

legend("topleft", c("High risk", "Low risk"),bty="n",pch=19,col=c("#C6524A","#2874C5"),cex=1.2)

dev.off()

###Risk Status

color=as.vector(rt$DOD)

color[color==1]="#C6524A"

color[color==0]="#2874C5"

pdf(file="GSE32894_survStat_Figure3G.pdf",width = 10,height = 4)

plot(rt$DOD.time,

pch=19,

xlab="Patients (increasing risk socre)",

ylab="Survival time (years)",

col=color)

legend("topleft", c("DOD", "Censored"),bty="n",pch=19,col=c("#C6524A","#2874C5"),cex=1.2)

abline(v=lowLength,lty=2)

dev.off()

#Risk Heatmap#

GSE32894 <- GSE32894[rownames(model),]

annotation <- data.frame(type=rt[,ncol(rt)])

annotation$type <- gsub("low risk","Low",annotation$type)

annotation$type <- gsub("high risk","High",annotation$type)

rownames(annotation)=rownames(rt)

colnames(GSE32894)=rownames(rt)

pdf(file="GSE32894_heatmap_Figure3G.pdf",width = 10,height = 4)

pheatmap(GSE32894,

scale = "column",

annotation=annotation,

cluster_cols = FALSE,

fontsize_row=11,

show_colnames = F,

fontsize_col=3,

color = colorRampPalette(c("#2874C5", "white", "#C6524A"))(50) )

dev.off()

#########ROC **(Figure 3D)** #################

library(survivalROC)

library(timeROC)

GSE32894data$id <- rownames(GSE32894data)

data1 <- GSE32894data[,c(1,2,12,13)]

data1$DOD.time.Year <- data1$DOD.time/12

predict_3_year<- 3*1

predict_5_year<- 5*1

table(subset(data1,data1$DOD.time.Year<3)$DOD)

ROC<-timeROC(T=data1$DOD.time.Year,delta=data1$DOD==1,

marker=data1$score,cause=1,

weighting="marginal",

times=c(predict_3_year,predict_5_year),ROC=TRUE)

pdf("GSE32894_Figure3D.pdf")

plot(ROC,time=predict_3_year,col="#2874C5",title=FALSE,lwd=3)

plot(ROC,time=predict_5_year,col="#C6524A",add=TRUE,title=FALSE,lwd=3)

legend("bottomright",

c(paste("AUC of 3 year survival: ",round(ROC$AUC[1],3)),

paste("AUC of 5 year survival: ",round(ROC$AUC[2],3))),col=c("#2874C5","#C6524A"),lwd=3)

dev.off()

#####Violin plots **(Figure 4A-C)**################

###Stage **(Figure 4A)**

GSE32894data$X1.TUMOR_STAGE<-gsub("T2a","T2",GSE32894data$X1.TUMOR_STAGE)

GSE32894data$X1.TUMOR_STAGE<-gsub("T2b","T2",GSE32894data$X1.TUMOR_STAGE)

GSE32894data$X1.TUMOR_STAGE<-gsub("T3","T3-4",GSE32894data$X1.TUMOR_STAGE)

GSE32894data$X1.TUMOR_STAGE<-gsub("T3-4b","T3-4",GSE32894data$X1.TUMOR_STAGE)

GSE32894data$X1.TUMOR_STAGE<-gsub("T3b","T3-4",GSE32894data$X1.TUMOR_STAGE)

GSE32894data$X1.TUMOR_STAGE<-gsub("T4a","T3-4",GSE32894data$X1.TUMOR_STAGE)

GSE32894data$X1.TUMOR_STAGE<-as.factor(GSE32894data$X1.TUMOR_STAGE)

GSE32894data$X1.TUMOR_STAGE<- factor(GSE32894data$X1.TUMOR_STAGE,levels = c("Ta","T1","T2","T3-4"))

compare_means(score~ X1.TUMOR_STAGE, data = GSE32894data)

my_comparisons<-list( c("Ta", "T1"),

c("Ta", "T2"),

c("Ta", "T3-4"),

c("T1", "T2"),

c("T1", "T3-4"))

pdf("GSE32894_Figure4A.pdf",width=4.9,height=5,file = "D:/GSE32894_Figure4A.pdf")

ggplot(GSE32894data,

aes(x=X1.TUMOR_STAGE,y=score,fill=X1.TUMOR_STAGE)) +

geom_jitter(aes(color=X1.TUMOR_STAGE),alpha=0.5) + scale_color_brewer(palette = 'Set1',direction=-1) +

geom_violin(alpha=0,size=1.4) + theme_bw() +

ggtitle("GSE32894 cohort")+theme_bw()+ theme(legend.position="none", axis.text.x=element_text(colour="black",family="Times",size=14), axis.text.y=element_text(family="Times",size=14,face="plain"), axis.title.y=element_text(family="Times",size = 14,face="plain"), axis.title.x=element_text(family="Times",size = 14,face="plain"), plot.title = element_text(family="Times",size=15,face="bold",hjust = 0.5),panel.grid.major = element_blank(), panel.grid.minor = element_blank())+

ylab("Risk score")+xlab("") +

stat_compare_means(comparisons = my_comparisons)+

stat_compare_means(label.y = 5,label.x = 1)

dev.off()

###grade **(Figure 4B)**

GSE32894data$X1.TUMOR_GRADE<-as.factor(GSE32894data$X1.TUMOR_GRADE)

GSE32894data <- GSE32894data [-which(GSE32894data$X1.TUMOR_GRADE=="Gx"),]

compare_means(score~ X1.TUMOR_GRADE, data = GSE32894data)

my_comparisons<-list( c("G1", "G2"),

c("G1", "G3"),

c("G2", "G3"))

pdf("GEO32894_Figure4B.pdf",width=4.9,height=5,file = "D:/GEO32894_Figure4B.pdf")

ggplot(GSE32894data,

aes(x=X1.TUMOR_GRADE,y=score,fill=X1.TUMOR_GRADE)) +

geom_jitter(aes(color=X1.TUMOR_GRADE),alpha=0.5) + scale_color_brewer(palette = 'Set1',direction=-1) +

geom_violin(alpha=0,size=1.4) + theme_bw() +

ggtitle("GSE32894 cohort")+theme_bw()+ theme(legend.position="none", axis.text.x=element_text(colour="black",family="Times",size=14), axis.text.y=element_text(family="Times",size=14,face="plain"), axis.title.y=element_text(family="Times",size = 14,face="plain"), axis.title.x=element_text(family="Times",size = 14,face="plain"), plot.title = element_text(family="Times",size=15,face="bold",hjust = 0.5),panel.grid.major = element_blank(), panel.grid.minor = element_blank())+

ylab("Risk score")+xlab("") +

stat_compare_means(comparisons = my_comparisons)+

stat_compare_means(label.y = 5,label.x = 1)

dev.off()

###Molecular subtype **(Figure 4C)**

table(GSE32894data$X1.MOLECULAR_SUBTYPE)

GSE32894data$X1.MOLECULAR_SUBTYPE.<-as.factor(GSE32894data$X1.MOLECULAR_SUBTYPE)

GSE32894data$X1.MOLECULAR_SUBTYPE<-gsub("MS2a.1","MS2a",GSE32894data$X1.MOLECULAR_SUBTYPE)

GSE32894data$X1.MOLECULAR_SUBTYPE<-gsub("MS2a.2","MS2a",GSE32894data$X1.MOLECULAR_SUBTYPE)

GSE32894data$X1.MOLECULAR_SUBTYPE<-gsub("MS2b.1","MS2b",GSE32894data$X1.MOLECULAR_SUBTYPE)

GSE32894data$X1.MOLECULAR_SUBTYPE<-gsub("MS2b2.1","MS2b2",GSE32894data$X1.MOLECULAR_SUBTYPE)

GSE32894data$X1.MOLECULAR_SUBTYPE<-gsub("MS2b2.2","MS2b2",GSE32894data$X1.MOLECULAR_SUBTYPE)

table(GSE32894data$X1.MOLECULAR_SUBTYPE)

compare_means(score~X1.MOLECULAR_SUBTYPE, data = GSE32894data)

my_comparisons<-list( c("MS1b", "MS2b"),

c("MS1b", "MS2b2"),

c("MS1b", "MS2a"),

c("MS2b", "MS1a"),

c("MS2b", "MS2b2"),

c("MS1a", "MS2b2"),

c("MS1a", "MS2a"),

c("MS2b2", "MS2a"))

pdf("GEO32894_Figure4C.pdf",width=4.9,height=5,file = "D:/GEO32894_Figure4C.pdf")

ggplot(GSE32894data,

aes(x=X1.MOLECULAR_SUBTYPE,y=score,fill=X1.MOLECULAR_SUBTYPE)) +

geom_jitter(aes(color=X1.MOLECULAR_SUBTYPE),alpha=0.5) + scale_color_brewer(palette = 'Set1',direction=-1) +

geom_violin(alpha=0,size=1.4) + theme_bw() +

ggtitle("GSE32894 cohort")+theme_bw()+ theme(legend.position="none", axis.text.x=element_text(colour="black",family="Times",size=14), axis.text.y=element_text(family="Times",size=14,face="plain"), axis.title.y=element_text(family="Times",size = 14,face="plain"), axis.title.x=element_text(family="Times",size = 14,face="plain"), plot.title = element_text(family="Times",size=15,face="bold",hjust = 0.5),panel.grid.major = element_blank(), panel.grid.minor = element_blank())+

ylab("Risk score")+xlab("") +

stat_compare_means(comparisons = my_comparisons)+

stat_compare_means(label.y = 5,label.x = 1)

dev.off()

##########Straified Analysis **(Figure 5)**####

##less than 65 **(Figure 5A)**

library(survival)

library("survminer")

rt=read.table("less than 65.txt",header=T,sep="\t")

rt$futime=rt$futime*12

diff=survdiff(Surv(futime, fustat) ~risk,data = rt)

pValue=1-pchisq(diff$chisq,df=1)

pValue=signif(pValue,4)

pValue=format(pValue, scientific = TRUE)

fit <- survfit(Surv(futime, fustat) ~ risk, data = rt)

p.val = 1 - pchisq(data.survdiff$chisq,length(data.survdiff$n) - 1)

p.val

HR = (data.survdiff$obs[1]/data.survdiff$exp[1])/(data.survdiff$obs[2]/data.survdiff$exp[2])

up95 = exp(log(HR) + qnorm(0.975)*sqrt(1/data.survdiff$exp[2]+1/data.survdiff$exp[1]))

low95 = exp(log(HR) - qnorm(0.975)*sqrt(1/data.survdiff$exp[2]+1/data.survdiff$exp[1]))

CI=paste("95%CI: ",paste(round(low95,2),seq=" - ",round(up95,2), collapse = ","),seq="")

HR<-paste("Hazard Ratio = ",round(HR,2),seq="")

pdf("less than 65_Figure5A.pdf",width=6.23,height=6.57,file = "D:/ less than 65_Figure5A.pdf")

ggsurvplot(fit, size=2.2,

legend.labs = c("High risk","Low risk"),

pval=paste0("p=",pValue),

conf.int=F,risk.table=T,risk.table.col="black" ,

pval.size=5,pval.coord= c(-0.1,0.1),

legend=c(0.15,0.3),

legend.title="",

font.legend=13,xlab="Months",ggtheme = theme_survminer(),

test.for.trend = T,linetype = 1,cumevents = F,censor.shape=3,

censor.size=3,

pval.method.size=10,pval.method=T,pval.method.coord= c(0,0.15),

risk.table.fontsize=4, font.x=18, font.y=18,

tables.y.text=T, palette = c("#C6524A","#2874C5"))

dev.off()

##more and than 65 **(Figure 5B)**

library(survival)

library("survminer")

rt=read.table("more and than 65.txt",header=T,sep="\t")

rt$futime=rt$futime*12

diff=survdiff(Surv(futime, fustat) ~risk,data = rt)

pValue=1-pchisq(diff$chisq,df=1)

pValue=signif(pValue,4)

pValue=format(pValue, scientific = TRUE)

fit <- survfit(Surv(futime, fustat) ~ risk, data = rt)

p.val = 1 - pchisq(data.survdiff$chisq,length(data.survdiff$n) - 1)

p.val

HR = (data.survdiff$obs[1]/data.survdiff$exp[1])/(data.survdiff$obs[2]/data.survdiff$exp[2])

up95 = exp(log(HR) + qnorm(0.975)*sqrt(1/data.survdiff$exp[2]+1/data.survdiff$exp[1]))

low95 = exp(log(HR) - qnorm(0.975)*sqrt(1/data.survdiff$exp[2]+1/data.survdiff$exp[1]))

CI=paste("95%CI: ",paste(round(low95,2),seq=" - ",round(up95,2), collapse = ","),seq="")

HR<-paste("Hazard Ratio = ",round(HR,2),seq="")

pdf("more and than 65_Figure5B.pdf",width=6.23,height=6.57,file = "D:/ more and than 65_Figure5B.pdf")

ggsurvplot(fit, size=2.2,

legend.labs = c("High risk","Low risk"),

pval=paste0("p=",pValue),

conf.int=F,risk.table=T,risk.table.col="black" ,

pval.size=5,pval.coord= c(-0.1,0.1),

legend=c(0.15,0.3),

legend.title="",

font.legend=13,xlab="Months",ggtheme = theme_survminer(),

test.for.trend = T,linetype = 1,cumevents = F,censor.shape=3,

censor.size=3,

pval.method.size=10,pval.method=T,pval.method.coord= c(0,0.15),

risk.table.fontsize=4, font.x=18, font.y=18,

tables.y.text=T, palette = c("#C6524A","#2874C5"))

dev.off()

##female **(Figure 5C)**

library(survival)

library("survminer")

rt=read.table("female.txt",header=T,sep="\t")

rt$futime=rt$futime*12

diff=survdiff(Surv(futime, fustat) ~risk,data = rt)

pValue=1-pchisq(diff$chisq,df=1)

pValue=signif(pValue,4)

pValue=format(pValue, scientific = TRUE)

fit <- survfit(Surv(futime, fustat) ~ risk, data = rt)

p.val = 1 - pchisq(data.survdiff$chisq,length(data.survdiff$n) - 1)

p.val

HR = (data.survdiff$obs[1]/data.survdiff$exp[1])/(data.survdiff$obs[2]/data.survdiff$exp[2])

up95 = exp(log(HR) + qnorm(0.975)*sqrt(1/data.survdiff$exp[2]+1/data.survdiff$exp[1]))

low95 = exp(log(HR) - qnorm(0.975)*sqrt(1/data.survdiff$exp[2]+1/data.survdiff$exp[1]))

CI=paste("95%CI: ",paste(round(low95,2),seq=" - ",round(up95,2), collapse = ","),seq="")

HR<-paste("Hazard Ratio = ",round(HR,2),seq="")

pdf("female_Figure5C.pdf",width=6.23,height=6.57,file = "D:/female_Figure5C.pdf")

ggsurvplot(fit, size=2.2,

legend.labs = c("High risk","Low risk"),

pval=paste0("p=",pValue),

conf.int=F,risk.table=T,risk.table.col="black" ,

pval.size=5,pval.coord= c(-0.1,0.1),

legend=c(0.15,0.3),

legend.title="",

font.legend=13,xlab="Months",ggtheme = theme_survminer(),

test.for.trend = T,linetype = 1,cumevents = F,censor.shape=3,

censor.size=3,

pval.method.size=10,pval.method=T,pval.method.coord= c(0,0.15),

risk.table.fontsize=4, font.x=18, font.y=18,

tables.y.text=T, palette = c("#C6524A","#2874C5"))

dev.off()

##male **(Figure 5D)**

library(survival)

library("survminer")

rt=read.table("male.txt",header=T,sep="\t")

rt$futime=rt$futime*12

diff=survdiff(Surv(futime, fustat) ~risk,data = rt)

pValue=1-pchisq(diff$chisq,df=1)

pValue=signif(pValue,4)

pValue=format(pValue, scientific = TRUE)

fit <- survfit(Surv(futime, fustat) ~ risk, data = rt)

p.val = 1 - pchisq(data.survdiff$chisq,length(data.survdiff$n) - 1)

p.val

HR = (data.survdiff$obs[1]/data.survdiff$exp[1])/(data.survdiff$obs[2]/data.survdiff$exp[2])

up95 = exp(log(HR) + qnorm(0.975)*sqrt(1/data.survdiff$exp[2]+1/data.survdiff$exp[1]))

low95 = exp(log(HR) - qnorm(0.975)*sqrt(1/data.survdiff$exp[2]+1/data.survdiff$exp[1]))

CI=paste("95%CI: ",paste(round(low95,2),seq=" - ",round(up95,2), collapse = ","),seq="")

HR<-paste("Hazard Ratio = ",round(HR,2),seq="")

pdf("male_Figure5D.pdf",width=6.23,height=6.57,file = "D:/male_Figure5D.pdf")

ggsurvplot(fit, size=2.2,

legend.labs = c("High risk","Low risk"),

pval=paste0("p=",pValue),

conf.int=F,risk.table=T,risk.table.col="black" ,

pval.size=5,pval.coord= c(-0.1,0.1),

legend=c(0.15,0.3),

legend.title="",

font.legend=13,xlab="Months",ggtheme = theme_survminer(),

test.for.trend = T,linetype = 1,cumevents = F,censor.shape=3,

censor.size=3,

pval.method.size=10,pval.method=T,pval.method.coord= c(0,0.15),

risk.table.fontsize=4, font.x=18, font.y=18,

tables.y.text=T, palette = c("#C6524A","#2874C5"))

dev.off()

##low grade **(Figure 5E)**

library(survival)

library("survminer")

rt=read.table("low grade.txt",header=T,sep="\t")

rt$futime=rt$futime*12

diff=survdiff(Surv(futime, fustat) ~risk,data = rt)

pValue=1-pchisq(diff$chisq,df=1)

pValue=signif(pValue,4)

pValue=format(pValue, scientific = TRUE)

fit <- survfit(Surv(futime, fustat) ~ risk, data = rt)

p.val = 1 - pchisq(data.survdiff$chisq,length(data.survdiff$n) - 1)

p.val

HR = (data.survdiff$obs[1]/data.survdiff$exp[1])/(data.survdiff$obs[2]/data.survdiff$exp[2])

up95 = exp(log(HR) + qnorm(0.975)*sqrt(1/data.survdiff$exp[2]+1/data.survdiff$exp[1]))

low95 = exp(log(HR) - qnorm(0.975)*sqrt(1/data.survdiff$exp[2]+1/data.survdiff$exp[1]))

CI=paste("95%CI: ",paste(round(low95,2),seq=" - ",round(up95,2), collapse = ","),seq="")

HR<-paste("Hazard Ratio = ",round(HR,2),seq="")

pdf("low grade_Figure5E.pdf",width=6.23,height=6.57,file = "D:/low grade_Figure5E.pdf")

ggsurvplot(fit, size=2.2,

legend.labs = c("High risk","Low risk"),

pval=paste0("p=",pValue),

conf.int=F,risk.table=T,risk.table.col="black" ,

pval.size=5,pval.coord= c(-0.1,0.1),

legend=c(0.15,0.3),

legend.title="",

font.legend=13,xlab="Months",ggtheme = theme_survminer(),

test.for.trend = T,linetype = 1,cumevents = F,censor.shape=3,

censor.size=3,

pval.method.size=10,pval.method=T,pval.method.coord= c(0,0.15),

risk.table.fontsize=4, font.x=18, font.y=18,

tables.y.text=T, palette = c("#C6524A","#2874C5"))

dev.off()

##high grade **(Figure 5F)**

library(survival)

library("survminer")

rt=read.table("high grade.txt",header=T,sep="\t")

rt$futime=rt$futime*12

diff=survdiff(Surv(futime, fustat) ~risk,data = rt)

pValue=1-pchisq(diff$chisq,df=1)

pValue=signif(pValue,4)

pValue=format(pValue, scientific = TRUE)

fit <- survfit(Surv(futime, fustat) ~ risk, data = rt)

p.val = 1 - pchisq(data.survdiff$chisq,length(data.survdiff$n) - 1)

p.val

HR = (data.survdiff$obs[1]/data.survdiff$exp[1])/(data.survdiff$obs[2]/data.survdiff$exp[2])

up95 = exp(log(HR) + qnorm(0.975)*sqrt(1/data.survdiff$exp[2]+1/data.survdiff$exp[1]))

low95 = exp(log(HR) - qnorm(0.975)*sqrt(1/data.survdiff$exp[2]+1/data.survdiff$exp[1]))

CI=paste("95%CI: ",paste(round(low95,2),seq=" - ",round(up95,2), collapse = ","),seq="")

HR<-paste("Hazard Ratio = ",round(HR,2),seq="")

pdf("high grade_Figure5F.pdf",width=6.23,height=6.57,file = "D:/high grade_Figure5F.pdf")

ggsurvplot(fit, size=2.2,

legend.labs = c("High risk","Low risk"),

pval=paste0("p=",pValue),

conf.int=F,risk.table=T,risk.table.col="black" ,

pval.size=5,pval.coord= c(-0.1,0.1),

legend=c(0.15,0.3),

legend.title="",

font.legend=13,xlab="Months",ggtheme = theme_survminer(),

test.for.trend = T,linetype = 1,cumevents = F,censor.shape=3,

censor.size=3,

pval.method.size=10,pval.method=T,pval.method.coord= c(0,0.15),

risk.table.fontsize=4, font.x=18, font.y=18,

tables.y.text=T, palette = c("#C6524A","#2874C5"))

dev.off()

##Ta-T1 **(Figure 5G)**

library(survival)

library("survminer")

rt=read.table("Ta-T1.txt",header=T,sep="\t")

rt$futime=rt$futime*12

diff=survdiff(Surv(futime, fustat) ~risk,data = rt)

pValue=1-pchisq(diff$chisq,df=1)

pValue=signif(pValue,4)

pValue=format(pValue, scientific = TRUE)

fit <- survfit(Surv(futime, fustat) ~ risk, data = rt)

p.val = 1 - pchisq(data.survdiff$chisq,length(data.survdiff$n) - 1)

p.val

HR = (data.survdiff$obs[1]/data.survdiff$exp[1])/(data.survdiff$obs[2]/data.survdiff$exp[2])

up95 = exp(log(HR) + qnorm(0.975)*sqrt(1/data.survdiff$exp[2]+1/data.survdiff$exp[1]))

low95 = exp(log(HR) - qnorm(0.975)*sqrt(1/data.survdiff$exp[2]+1/data.survdiff$exp[1]))

CI=paste("95%CI: ",paste(round(low95,2),seq=" - ",round(up95,2), collapse = ","),seq="")

HR<-paste("Hazard Ratio = ",round(HR,2),seq="")

pdf("Ta-T1_Figure5G.pdf",width=6.23,height=6.57,file = "D:/ Ta-T1_Figure5G.pdf")

ggsurvplot(fit, size=2.2,

legend.labs = c("High risk","Low risk"),

pval=paste0("p=",pValue),

conf.int=F,risk.table=T,risk.table.col="black" ,

pval.size=5,pval.coord= c(-0.1,0.1),

legend=c(0.15,0.3),

legend.title="",

font.legend=13,xlab="Months",ggtheme = theme_survminer(),

test.for.trend = T,linetype = 1,cumevents = F,censor.shape=3,

censor.size=3,

pval.method.size=10,pval.method=T,pval.method.coord= c(0,0.15),

risk.table.fontsize=4, font.x=18, font.y=18,

tables.y.text=T, palette = c("#C6524A","#2874C5"))

dev.off()

##T2-T4 **(Figure 5H)**

library(survival)

library("survminer")

rt=read.table("T2-T4.txt",header=T,sep="\t")

rt$futime=rt$futime*12

diff=survdiff(Surv(futime, fustat) ~risk,data = rt)

pValue=1-pchisq(diff$chisq,df=1)

pValue=signif(pValue,4)

pValue=format(pValue, scientific = TRUE)

fit <- survfit(Surv(futime, fustat) ~ risk, data = rt)

p.val = 1 - pchisq(data.survdiff$chisq,length(data.survdiff$n) - 1)

p.val

HR = (data.survdiff$obs[1]/data.survdiff$exp[1])/(data.survdiff$obs[2]/data.survdiff$exp[2])

up95 = exp(log(HR) + qnorm(0.975)*sqrt(1/data.survdiff$exp[2]+1/data.survdiff$exp[1]))

low95 = exp(log(HR) - qnorm(0.975)*sqrt(1/data.survdiff$exp[2]+1/data.survdiff$exp[1]))

CI=paste("95%CI: ",paste(round(low95,2),seq=" - ",round(up95,2), collapse = ","),seq="")

HR<-paste("Hazard Ratio = ",round(HR,2),seq="")

pdf("T2-T4_Figure5H.pdf",width=6.23,height=6.57,file = "D:/ T2-T4_Figure5H.pdf")

ggsurvplot(fit, size=2.2,

legend.labs = c("High risk","Low risk"),

pval=paste0("p=",pValue),

conf.int=F,risk.table=T,risk.table.col="black" ,

pval.size=5,pval.coord= c(-0.1,0.1),

legend=c(0.15,0.3),

legend.title="",

font.legend=13,xlab="Months",ggtheme = theme_survminer(),

test.for.trend = T,linetype = 1,cumevents = F,censor.shape=3,

censor.size=3,

pval.method.size=10,pval.method=T,pval.method.coord= c(0,0.15),

risk.table.fontsize=4, font.x=18, font.y=18,

tables.y.text=T, palette = c("#C6524A","#2874C5"))

dev.off()

##N0 **(Figure 5I)**

library(survival)

library("survminer")

rt=read.table("N0.txt",header=T,sep="\t")

rt$futime=rt$futime*12

diff=survdiff(Surv(futime, fustat) ~risk,data = rt)

pValue=1-pchisq(diff$chisq,df=1)

pValue=signif(pValue,4)

pValue=format(pValue, scientific = TRUE)

fit <- survfit(Surv(futime, fustat) ~ risk, data = rt)

p.val = 1 - pchisq(data.survdiff$chisq,length(data.survdiff$n) - 1)

p.val

HR = (data.survdiff$obs[1]/data.survdiff$exp[1])/(data.survdiff$obs[2]/data.survdiff$exp[2])

up95 = exp(log(HR) + qnorm(0.975)*sqrt(1/data.survdiff$exp[2]+1/data.survdiff$exp[1]))

low95 = exp(log(HR) - qnorm(0.975)*sqrt(1/data.survdiff$exp[2]+1/data.survdiff$exp[1]))

CI=paste("95%CI: ",paste(round(low95,2),seq=" - ",round(up95,2), collapse = ","),seq="")

HR<-paste("Hazard Ratio = ",round(HR,2),seq="")

pdf("N0_Figure5I.pdf",width=6.23,height=6.57,file = "D:/ N0_Figure5I.pdf")

ggsurvplot(fit, size=2.2,

legend.labs = c("High risk","Low risk"),

pval=paste0("p=",pValue),

conf.int=F,risk.table=T,risk.table.col="black" ,

pval.size=5,pval.coord= c(-0.1,0.1),

legend=c(0.15,0.3),

legend.title="",

font.legend=13,xlab="Months",ggtheme = theme_survminer(),

test.for.trend = T,linetype = 1,cumevents = F,censor.shape=3,

censor.size=3,

pval.method.size=10,pval.method=T,pval.method.coord= c(0,0.15),

risk.table.fontsize=4, font.x=18, font.y=18,

tables.y.text=T, palette = c("#C6524A","#2874C5"))

dev.off()

##N+**(Figure 5J)**

library(survival)

library("survminer")

rt=read.table("N+.txt",header=T,sep="\t")

rt$futime=rt$futime*12

diff=survdiff(Surv(futime, fustat) ~risk,data = rt)

pValue=1-pchisq(diff$chisq,df=1)

pValue=signif(pValue,4)

pValue=format(pValue, scientific = TRUE)

fit <- survfit(Surv(futime, fustat) ~ risk, data = rt)

p.val = 1 - pchisq(data.survdiff$chisq,length(data.survdiff$n) - 1)

p.val

HR = (data.survdiff$obs[1]/data.survdiff$exp[1])/(data.survdiff$obs[2]/data.survdiff$exp[2])

up95 = exp(log(HR) + qnorm(0.975)*sqrt(1/data.survdiff$exp[2]+1/data.survdiff$exp[1]))

low95 = exp(log(HR) - qnorm(0.975)*sqrt(1/data.survdiff$exp[2]+1/data.survdiff$exp[1]))

CI=paste("95%CI: ",paste(round(low95,2),seq=" - ",round(up95,2), collapse = ","),seq="")

HR<-paste("Hazard Ratio = ",round(HR,2),seq="")

pdf("N+_Figure5J.pdf",width=6.23,height=6.57,file = "D:/ N+_Figure5J.pdf")

ggsurvplot(fit, size=2.2,

legend.labs = c("High risk","Low risk"),

pval=paste0("p=",pValue),

conf.int=F,risk.table=T,risk.table.col="black" ,

pval.size=5,pval.coord= c(-0.1,0.1),

legend=c(0.15,0.3),

legend.title="",

font.legend=13,xlab="Months",ggtheme = theme_survminer(),

test.for.trend = T,linetype = 1,cumevents = F,censor.shape=3,

censor.size=3,

pval.method.size=10,pval.method=T,pval.method.coord= c(0,0.15),

risk.table.fontsize=4, font.x=18, font.y=18,

tables.y.text=T, palette = c("#C6524A","#2874C5"))

dev.off()

##no progression **(Figure 5K)**

library(survival)

library("survminer")

rt=read.table("no progression.txt",header=T,sep="\t")

rt$futime=rt$futime*12

diff=survdiff(Surv(futime, fustat) ~risk,data = rt)

pValue=1-pchisq(diff$chisq,df=1)

pValue=signif(pValue,4)

pValue=format(pValue, scientific = TRUE)

fit <- survfit(Surv(futime, fustat) ~ risk, data = rt)

p.val = 1 - pchisq(data.survdiff$chisq,length(data.survdiff$n) - 1)

p.val

HR = (data.survdiff$obs[1]/data.survdiff$exp[1])/(data.survdiff$obs[2]/data.survdiff$exp[2])

up95 = exp(log(HR) + qnorm(0.975)*sqrt(1/data.survdiff$exp[2]+1/data.survdiff$exp[1]))

low95 = exp(log(HR) - qnorm(0.975)*sqrt(1/data.survdiff$exp[2]+1/data.survdiff$exp[1]))

CI=paste("95%CI: ",paste(round(low95,2),seq=" - ",round(up95,2), collapse = ","),seq="")

HR<-paste("Hazard Ratio = ",round(HR,2),seq="")

pdf("no progression_Figure5K.pdf",width=6.23,height=6.57,file = "D:/ no progression_Figure5K.pdf")

ggsurvplot(fit, size=2.2,

legend.labs = c("High risk","Low risk"),

pval=paste0("p=",pValue),

conf.int=F,risk.table=T,risk.table.col="black" ,

pval.size=5,pval.coord= c(-0.1,0.1),

legend=c(0.15,0.3),

legend.title="",

font.legend=13,xlab="Months",ggtheme = theme_survminer(),

test.for.trend = T,linetype = 1,cumevents = F,censor.shape=3,

censor.size=3,

pval.method.size=10,pval.method=T,pval.method.coord= c(0,0.15),

risk.table.fontsize=4, font.x=18, font.y=18,

tables.y.text=T, palette = c("#C6524A","#2874C5"))

dev.off()

##Progression **(Figure 5L)**

library(survival)

library("survminer")

rt=read.table("Progresssion.txt",header=T,sep="\t")

rt$futime=rt$futime*12

diff=survdiff(Surv(futime, fustat) ~risk,data = rt)

pValue=1-pchisq(diff$chisq,df=1)

pValue=signif(pValue,4)

pValue=format(pValue, scientific = TRUE)

fit <- survfit(Surv(futime, fustat) ~ risk, data = rt)

p.val = 1 - pchisq(data.survdiff$chisq,length(data.survdiff$n) - 1)

p.val

HR = (data.survdiff$obs[1]/data.survdiff$exp[1])/(data.survdiff$obs[2]/data.survdiff$exp[2])

up95 = exp(log(HR) + qnorm(0.975)*sqrt(1/data.survdiff$exp[2]+1/data.survdiff$exp[1]))

low95 = exp(log(HR) - qnorm(0.975)*sqrt(1/data.survdiff$exp[2]+1/data.survdiff$exp[1]))

CI=paste("95%CI: ",paste(round(low95,2),seq=" - ",round(up95,2), collapse = ","),seq="")

HR<-paste("Hazard Ratio = ",round(HR,2),seq="")

pdf("Progresssion_Figure5L.pdf",width=6.23,height=6.57,file = "D:/ Progresssion_Figure5L.pdf")

ggsurvplot(fit, size=2.2,

legend.labs = c("High risk","Low risk"),

pval=paste0("p=",pValue),

conf.int=F,risk.table=T,risk.table.col="black" ,

pval.size=5,pval.coord= c(-0.1,0.1),

legend=c(0.15,0.3),

legend.title="",

font.legend=13,xlab="Months",ggtheme = theme_survminer(),

test.for.trend = T,linetype = 1,cumevents = F,censor.shape=3,

censor.size=3,

pval.method.size=10,pval.method=T,pval.method.coord= c(0,0.15),

risk.table.fontsize=4, font.x=18, font.y=18,

tables.y.text=T, palette = c("#C6524A","#2874C5"))

dev.off()

########Difference Analysis **(Figure 6)**#####

library(limma)

library("impute")

geo_data<-read.table("classification.txt",sep="\t",header=T)

geo_data<-as.matrix(geo_data)

rownames(geo_data)=geo_data[,1]

geo_exp<-geo_data[,2:ncol(geo_data)]

dimnames<-list(rownames(geo_exp),colnames(geo_exp))

geo_exp<-matrix(as.numeric(as.matrix(geo_exp)),nrow=nrow(geo_exp),dimnames=dimnames)

mat=impute.knn(geo_exp)

geo_data=mat$data

geo_data=avereps(geo_data)

class <- c(rep("normal",112),rep("treatment",112))

design <- model.matrix(~0+factor(class))

colnames(design) <- c("normal","treatment")

fit <- lmFit(geo_data,design)

cont.matrix<-makeContrasts(treatment-normal,levels=design)

fit1 <- contrasts.fit(fit, cont.matrix)

fit1 <- eBayes(fit1)

allgene<-topTable(fit1,adjust='fdr',number=100000)

write.table(allgene,"allgene.xls",sep="\t",quote=F)

normaldata<-allgene[order(allgene$logFC),]

normaldata1<-rbind(Gene=colnames(normaldata),normaldata)

write.table(normaldata,"normaldata.txt",sep="\t",quote=F,col.names=F)

diffgene <- allgene[with(allgene, (abs(logFC)>=1 & adj.P.Val < 0.05 )), ]

write.table(diffgene,"diffgene.xls",sep="\t",quote=F)

Upgene <- allgene[with(allgene, (logFC>=1 & adj.P.Val < 0.05 )), ]

write.table(Upgene,"upgene.xls",sep="\t",quote=F)

Downgene <- allgene[with(allgene, (logFC<=(-1) & adj.P.Val < 0.05 )), ]

write.table(Downgene,"down.xls",sep="\t",quote=F)

diffexp=geo_data[rownames(diffgene),]

diffexp1=rbind(id=colnames(diffexp),diffexp)

write.table(diffexp1,"diffexp.txt",sep="\t",quote=F,col.names=F)

Figure 6A Volcano was visualized by Sangerbox8

####### Enrichment Analysis##

##up **(Figure 6C)**

library("org.Hs.eg.db")

rt=read.table("up.txt",sep="\t",check.names=F,header=T)

genes=as.vector(rt[,1])

entrezIDs <- mget(genes, org.Hs.egSYMBOL2EG, ifnotfound=NA)

entrezIDs <- as.character(entrezIDs)

out=cbind(rt,entrezID=entrezIDs)

write.table(out,file="up-id.txt",sep="\t",quote=F,row.names=F)

rt=read.table("up-id.txt",sep="\t",header=T,check.names=F)

rt=rt[is.na(rt[,"entrezID"])==F,]

gene=rt$entrezID

kk <- enrichGO(gene = gene,

OrgDb = org.Hs.eg.db,

pvalueCutoff =0.05,

qvalueCutoff = 0.05,

ont="all",

readable =T)

write.table(kk,file="up-GO.txt",sep="\t",quote=F,row.names = F)

pdf(file="Figure6C_GO.pdf",width = 10,height = 8)

dotplot(kk,showCategory = 10,split="ONTOLOGY") + facet_grid(ONTOLOGY~., scale='free')

dev.off()

##down **(Figure 6B)**

library("org.Hs.eg.db")

rt=read.table("down.txt",sep="\t",check.names=F,header=T)

genes=as.vector(rt[,1])

entrezIDs <- mget(genes, org.Hs.egSYMBOL2EG, ifnotfound=NA)

entrezIDs <- as.character(entrezIDs)

out=cbind(rt,entrezID=entrezIDs)

write.table(out,file="down-id.txt",sep="\t",quote=F,row.names=F)

rt=read.table("down-id.txt",sep="\t",header=T,check.names=F)

rt=rt[is.na(rt[,"entrezID"])==F,]

gene=rt$entrezID

kk <- enrichGO(gene = gene,

OrgDb = org.Hs.eg.db,

pvalueCutoff =0.05,

qvalueCutoff = 0.05,

ont="all",

readable =T)

write.table(kk,file="down-GO.txt",sep="\t",quote=F,row.names = F)

pdf(file="Figure6B_GO.pdf",width = 10,height = 8)

dotplot(kk,showCategory = 10,split="ONTOLOGY") + facet_grid(ONTOLOGY~., scale='free')

dev.off()

#####GSEA**(Figure 6D)**

Figure6D.GSEA analysis was performed by using GSEA_4.0.3

#########################GSE13507 Plots#################################

#####Survival Curve **(Figure 3B)**####

model<-read.delim("D:/multiCox.txt",header = 1)

rownames(model)<-model$id

model<-model %>% dplyr::select("coef")

model<-as.matrix(model)

GSE13507<-read.delim(file = "D:/GSE13507.txt",header = T,row.names = 1)

clinicaldata<-read.delim(file = "D:/GSE13507clinical.txt",header = T,row.names = 1)

GSE13507<-log2(GSE13507+1)

GSE13507<-GSE13507[,rownames(clinicaldata)]

data<-t(GSE13507)

GSE13507data<-clinicaldata

nall<-nrow(data)

RT<-data

classifier<-matrix(data=0,ncol=nall,nrow=(dim(model)[1]+1))

t<-t(RT)

intersect(rownames(model),rownames(GSE13507))

colnames(classifier)<-colnames(t)

for(j in 1:nall)

for(i in 1:dim(model)[1])

{classifier[i+1,j]=classifier[i,j]+model[i]*t[as.matrix(rownames(model))[i],j]}

classifier[dim(model)[1]+1,]

allscore<-classifier[dim(model)[1]+1,]

GSE13507data$score<-allscore

head(GSE13507data)

GSE13507data$CANCER.SPECIFIC.SURVIVAL<-as.factor(GSE13507data$CANCER.SPECIFIC.SURVIVAL)

GSE13507data$NEW_group=ifelse(GSE13507data$score > median(GSE13507data$score),'high risk','low risk')

table(GSE13507data$NEW_group)

fit <- survfit(Surv(GSE13507data$SURVIVAL.MONTH, GSE13507data$CANCER.SPECIFIC.SURVIVAL=="1")~GSE13507data$NEW_group, data=GSE13507data)

fit

my.surv <-Surv(GSE13507data$SURVIVAL.MONTH, GSE13507data$CANCER.SPECIFIC.SURVIVAL=="1")

data.survdiff <- survdiff(my.surv~GSE13507data$NEW_group,data=GSE13507data)

p.val = 1 - pchisq(data.survdiff$chisq,length(data.survdiff$n) - 1)

p.val

HR = (data.survdiff$obs[1]/data.survdiff$exp[1])/(data.survdiff$obs[2]/data.survdiff$exp[2])

up95 = exp(log(HR) + qnorm(0.975)*sqrt(1/data.survdiff$exp[2]+1/data.survdiff$exp[1]))

low95 = exp(log(HR) - qnorm(0.975)*sqrt(1/data.survdiff$exp[2]+1/data.survdiff$exp[1]))

CI=paste("95%CI: ",paste(round(low95,2),seq=" - ",round(up95,2), collapse = ","),seq="")

HR<-paste("Hazard Ratio = ",round(HR,2),seq="")

pdf("GSE13507_Figure3B.pdf",width=6.23,height=6.57,file = "D:/GSE13507_Figure3B.pdf")

ggsurvplot(fit, size=2.2,

legend.labs = c("High risk","Low risk"),

pval=paste("p=",round(p.val,4)),

conf.int=F,risk.table=T,risk.table.col="black" ,

pval.size=5,pval.coord= c(-0.1,0.1),

legend=c(0.2,0.3),

legend.title="",

font.legend=13,xlab="Months",ggtheme = theme_survminer(),

test.for.trend = T,linetype = 1,cumevents = F,censor.shape=3,

censor.size=3,

pval.method.size=10,pval.method=T,pval.method.coord= c(0,0.15),

risk.table.fontsize=4, font.x=18, font.y=18,

tables.y.text=T, palette = c("#C6524A","#2874C5"))

dev.off()

#####Survival Riskplot **(Figure 3H)#######**

library(pheatmap

rt=GSE13507data[order(GSE13507data$score),]

###Risk Score

riskClass=rt[,"NEW_group"]

lowLength=length(riskClass[riskClass=="low risk"])

highLength=length(riskClass[riskClass=="high risk"])

line=rt[,"score"]

pdf(file="GSE13507_riskScore_Figure3H.pdf",width = 10,height = 4)

plot(line,

type="p",

pch=20,

xlab="Patients (increasing risk socre)",

ylab="Risk score",

col=c(rep("#2874C5",lowLength),

rep("#C6524A",highLength)))

abline(h=median(rt$riskScore),v=lowLength,lty=2)

legend("topleft", c("High risk", "Low risk"),bty="n",pch=19,col=c("#C6524A","#2874C5"),cex=1.2)

dev.off()

color=as.vector(rt$CANCER.SPECIFIC.SURVIVAL)

color[color==1]="#C6524A"

color[color==0]="#2874C5"

pdf(file="GSE13507_survStat_Figure3H.pdf",width = 10,height = 4)

plot(rt$SURVIVAL.MONTH,

pch=19,

xlab="Patients (increasing risk socre)",

ylab="Survival time (years)",

col=color)

legend("topleft", c("CSS", "Censored"),bty="n",pch=19,col=c("#C6524A","#2874C5"),cex=1.2)

abline(v=lowLength,lty=2)

dev.off()

###Risk Heatmap

GSE13507 <- GSE13507[rownames(model),]

annotation <- data.frame(type=rt[,ncol(rt)])

annotation$type <- gsub("low risk","Low",annotation$type)

annotation$type <- gsub("high risk","High",annotation$type)

rownames(annotation)=rownames(rt)

colnames(GSE13507)=rownames(rt)

pdf(file="GSE13507_heatmap_Figure3H.pdf",width = 10,height = 4)

pheatmap(GSE13507,

scale = "column",

annotation=annotation,

cluster_cols = FALSE,

fontsize_row=11,

show_colnames = F,

fontsize_col=3,

color = colorRampPalette(c("#2874C5", "white", "#C6524A"))(50) )

dev.off()

###########ROC **(Figure 3E)**#########

library(survivalROC)

library(timeROC)

GSE13507data$SURVIVAL.MONTH.Year <- GSE13507data$SURVIVAL.MONTH/12

predict_3_year<- 3*1

predict_5_year<- 5*1

ROC<-timeROC(T=GSE13507data$SURVIVAL.MONTH.Year,delta=GSE13507data$CANCER.SPECIFIC.SURVIVAL==1,

marker=GSE13507data$score,cause=1,

weighting="marginal",

times=c(predict_3_year,predict_5_year),ROC=TRUE)

pdf("GSE13507_ROC_Figure3E.pdf")

plot(ROC,time=predict_3_year,col="#2874C5",title=FALSE,lwd=3)

plot(ROC,time=predict_5_year,col="#C6524A",add=TRUE,title=FALSE,lwd=3)

legend("bottomright",

c(paste("AUC of 3 year survival: ",round(ROC$AUC[1],3)),

paste("AUC of 5 year survival: ",round(ROC$AUC[2],3))),col=c("#2874C5","#C6524A"),lwd=3)

dev.off()

#########Violin Plots **(Figure 4D-F)** ######

###Stage **(Figure 4D)**

GSE13507data$X1.STAGE<-gsub("T2a","T2",GSE13507data$X1.STAGE)

GSE13507data$X1.STAGE<-gsub("T2b","T2",GSE13507data$X1.STAGE)

GSE13507data$X1.STAGE<-gsub("T3a","T3",GSE13507data$X1.STAGE)

GSE13507data$X1.STAGE<-gsub("T3b","T3",GSE13507data$X1.STAGE)

GSE13507data$X1.STAGE<-gsub("T4a","T4",GSE13507data$X1.STAGE)

GSE13507data$T<-substring(GSE13507data$X1.STAGE,1,2)

table(GSE13507data$T)

GSE13507data$M<-substring(GSE13507data$X1.STAGE,5,6)

table(GSE13507data$M)

GSE13507data$N<-substring(GSE13507data$X1.STAGE,3,4)

table(GSE13507data$N)

GSE13507data$T<-as.factor(GSE13507data$T)

GSE13507data$T<- factor(GSE13507data$T,levels = c("Ta","T1","T2","T3","T4"))

compare_means(score~T, data = GSE13507data)

my_comparisons<-list( c("Ta", "T2"),

c("Ta", "T3"),

c("Ta", "T4"),

c("T1","T2"),

c("T1","T3"),

c("T1","T4"),

c("T2","T3"))

pdf("GSE13507_Figure4D.pdf",width=4.9,height=5,file = "D:/GSE13507_T_Figure4D.pdf")

ggplot(GSE13507data,

aes(x=T,y=score,fill=T)) +

geom_jitter(aes(color=T),alpha=0.5) + scale_color_brewer(palette = 'Set1',direction=-1) +

geom_violin(alpha=0,size=1.4) + theme_bw() +

ggtitle("GSE13507 cohort")+theme_bw()+ theme(legend.position="none", axis.text.x=element_text(colour="black",family="Times",size=14), axis.text.y=element_text(family="Times",size=14,face="plain"), axis.title.y=element_text(family="Times",size = 14,face="plain"), axis.title.x=element_text(family="Times",size = 14,face="plain"), plot.title = element_text(family="Times",size=15,face="bold",hjust = 0.5),panel.grid.major = element_blank(), panel.grid.minor = element_blank())+

ylab("Risk score")+xlab("") +

stat_compare_means(comparisons = my_comparisons)+

stat_compare_means(label.y = 12.5,label.x = 1)

dev.off()

#####grade **(Figure 4E)**

GSE13507data$X1.GRADE <- gsub("high",'High grade',GSE13507data$X1.GRADE)

GSE13507data$X1.GRADE <- gsub("low",'Low grade',GSE13507data$X1.GRADE)

GSE13507data$X1.GRADE<- factor(GSE13507data$X1.GRADE,levels = c("Low grade","High grade"))

compare_means(score~ X1.GRADE, data = GSE13507data)

my_comparisons<-list( c("Low grade", "High grade"))

pdf("GSE13507_Figure4E.pdf",width=4.9,height=5,file = "D:/GSE13507_grade_Figure4E.pdf")

ggplot(GSE13507data,

aes(x=X1.GRADE,y=score,fill=X1.GRADE)) +

geom_jitter(aes(color=X1.GRADE),alpha=0.5) + scale_color_brewer(palette = 'Set1',direction=-1) +

geom_violin(alpha=0,size=1.4) + theme_bw() +

ggtitle("GSE13507 cohort")+theme_bw()+ theme(legend.position="none", axis.text.x=element_text(colour="black",family="Times",size=14), axis.text.y=element_text(family="Times",size=14,face="plain"), axis.title.y=element_text(family="Times",size = 14,face="plain"), axis.title.x=element_text(family="Times",size = 14,face="plain"), plot.title = element_text(family="Times",size=15,face="bold",hjust = 0.5),panel.grid.major = element_blank(), panel.grid.minor = element_blank())+

ylab("Risk score")+xlab("") +

stat_compare_means(comparisons = my_comparisons)+

stat_compare_means(label.y = 12.5,label.x = 1)

dev.off()

###Progression **(Figure 4F)**

GSE13507data$X1.PROGRESSION<-as.factor(GSE13507data$X1.PROGRESSION)

GSE13507data$X1.PROGRESSION<- ifelse(GSE13507data$X1.PROGRESSION=='No',"No progression","Progression")

compare_means(score~ X1.PROGRESSION, data = GSE13507data)

my_comparisons<-list( c("No progression", " Progression"))

pdf("GSE13507_Figure4F.pdf",width=4.9,height=5,file = "D:/GSE13507_PROGRESSION_Figure4F.pdf")

ggplot(GSE13507data,

aes(x=X1.PROGRESSION,y=score,fill=X1.PROGRESSION)) +

geom_jitter(aes(color=X1.PROGRESSION),alpha=0.5) + scale_color_brewer(palette = 'Set1',direction=-1) +

geom_violin(alpha=0,size=1.4) + theme_bw() +

ggtitle("GSE13507 cohort")+theme_bw()+ theme(legend.position="none", axis.text.x=element_text(colour="black",family="Times",size=14), axis.text.y=element_text(family="Times",size=14,face="plain"), axis.title.y=element_text(family="Times",size = 14,face="plain"), axis.title.x=element_text(family="Times",size = 14,face="plain"), plot.title = element_text(family="Times",size=15,face="bold",hjust = 0.5),panel.grid.major = element_blank(), panel.grid.minor = element_blank())+

ylab("Risk score")+xlab("") +

stat_compare_means(comparisons = my_comparisons)+

stat_compare_means(label.y = 12.5,label.x = 1)

dev.off()

#########################TCGA Plots ####################################

######Survival Plot **(Figure 3C)**##

library(dplyr)

library(survival)

library(ggplot2)

library(survminer)

clinicaldata<-read.delim(file = "D:/TCGA_Clinical.txt",header = T,row.names = 1)

load("D:/blca_tpm.Rdata")

model<-read.delim("D:/multiCox.txt",header = 1)

rownames(model)<-model$id

model<-model %>% dplyr::select("coef")

model<-as.matrix(model)

blca_tpmlog<-log2(blca_tpm+1)

colnames(blca_tpmlog) <- substring(colnames(blca_tpmlog),1,12)

clinicaldatasec<- clinicaldata %>% dplyr::select("DSS","DSS.time")

clinicaldatasec<- clinicaldatasec[-which(clinicaldatasec$DSS=="#N/A"),]

clinicaldatasec$DSS<- factor(clinicaldatasec$DSS,levels = c(1,0))

clinicaldatasec<- clinicaldatasec[intersect(rownames(clinicaldatasec),colnames(blca_tpmlog)),]

blca_tpmlog<- blca_tpmlog[,intersect(rownames(clinicaldatasec),colnames(blca_tpmlog))]

RT<-as.matrix(t(blca_tpmlog))

nsampleslas<-nrow(RT)

nsampleslas

classifier<-matrix(data=0,ncol=nsampleslas,nrow=(dim(model)[1]+1))

t<-t(RT)

intersect(rownames(model),rownames(blca_tpmlog))

colnames(classifier)<-colnames(t)

for(j in 1:nsampleslas)

for(i in 1:dim(model)[1])

{classifier[i+1,j]=classifier[i,j]+model[i]*t[as.matrix(rownames(model))[i],j]}

classifier[dim(model)[1]+1,]

score<-as.data.frame( classifier[dim(model)[1]+1,])

clinicaldatasec$score<-score$`classifier[dim(model)[1] + 1, ]`

clinicaldatasec$CSS<-ifelse(clinicaldatasec$DSS=="1","1","0")

clinicaldatasec$CSS<-as.factor(clinicaldatasec$CSS)

clinicaldatasec$NEW_group=ifelse(clinicaldatasec$score > median(clinicaldatasec$score),'high risk','low risk')

table(clinicaldatasec$NEW_group)

fit <- survfit(Surv(clinicaldatasec$DSS.time,

clinicaldatasec$DSS==1)~clinicaldatasec$NEW_group,

data=clinicaldatasec)

fit

my.surv <-Surv(clinicaldatasec$DSS.time, clinicaldatasec$DSS=="1")

data.survdiff <- survdiff(my.surv~clinicaldatasec$NEW_group,data=clinicaldatasec)

p.val = 1 - pchisq(data.survdiff$chisq,length(data.survdiff$n) - 1)

p.val

HR = (data.survdiff$obs[1]/data.survdiff$exp[1])/(data.survdiff$obs[2]/data.survdiff$exp[2])

up95 = exp(log(HR) + qnorm(0.975)*sqrt(1/data.survdiff$exp[2]+1/data.survdiff$exp[1]))

low95 = exp(log(HR) - qnorm(0.975)*sqrt(1/data.survdiff$exp[2]+1/data.survdiff$exp[1]))

CI=paste("95%CI: ",paste(round(low95,2),seq=" - ",round(up95,2), collapse = ","),seq="")

HR<-paste("Hazard Ratio = ",round(HR,2),seq="")

pdf("TCGA_Figure3C.pdf",width=6.23,height=6.57,file = "D:/TCGA_Figure3C.pdf")

ggsurvplot(fit, size=2.2,

legend.labs = c("High risk","Low risk"),

pval=paste("p=",round(p.val,4)),

conf.int=F,risk.table=T,risk.table.col="black" ,

pval.size=5,pval.coord= c(-0.1,0.1),

legend=c(0.2,0.3),

legend.title="",

font.legend=13,xlab="Days",ggtheme = theme_survminer(),

test.for.trend = T,linetype = 1,cumevents = F,censor.shape=3,

censor.size=3,

pval.method.size=10,pval.method=T,pval.method.coord= c(0,0.15),

risk.table.fontsize=4, font.x=18, font.y=18,

tables.y.text=T, palette = c("#C6524A","#2874C5"))

dev.off()

#################Risk Plots **(Figure 3I)**####

library(pheatmap)

rt=clinicaldatasec[order(clinicaldatasec$score),]

###Risk Curve

riskClass=rt[,"NEW_group"]

lowLength=length(riskClass[riskClass=="low risk"])

highLength=length(riskClass[riskClass=="high risk"])

line=rt[,"score"]

#line[line>10]=10

pdf(file="TCGA_riskScore_Figure3I.pdf",width = 10,height = 4)

plot(line,

type="p",

pch=20,

xlab="Patients (increasing risk socre)",

ylab="Risk score",

col=c(rep("#2874C5",lowLength),

rep("#C6524A",highLength)))

abline(h=median(rt$riskScore),v=lowLength,lty=2)

legend("topleft", c("High risk", "Low risk"),bty="n",pch=19,col=c("#C6524A","#2874C5"),cex=1.2)

dev.off()

###Risk Status

color=as.vector(rt$DSS)

color[color==1]="#C6524A"

color[color==0]="#2874C5"

pdf(file="TCGA_survStat_Figure3I.pdf",width = 10,height = 4)

plot(rt$DSS.time,

pch=19,

xlab="Patients (increasing risk socre)",

ylab="Survival time (years)",

col=color)

legend("topleft", c("DSS", "Censored"),bty="n",pch=19,col=c("#C6524A","#2874C5"),cex=1.2)

abline(v=lowLength,lty=2)

dev.off()

###Risk Heatmap

blca_tpmlog_sec <- blca_tpmlog[rownames(model),]

annotation <- data.frame(type=rt[,ncol(rt)])

annotation$type <- gsub("low risk","low.risk",annotation$type)

annotation$type <- gsub("high risk","high.risk",annotation$type)

rownames(annotation)=rownames(rt)

colnames(blca_tpmlog_sec)=rownames(rt)

pdf(file="TCGA_heatmap_Figure3I.pdf",width = 10,height = 4)

pheatmap(blca_tpmlog_sec,

scale = "column",

annotation=annotation,

cluster_cols = FALSE,

fontsize_row=11,

show_colnames = F,

fontsize_col=3,

color = colorRampPalette(c("#C6524A", "white", "#2874C5"))(50) )

dev.off()

##########ROC **(Figure 3F)**#######

library(survivalROC)

clinicaldatasec$DSS.time.year <-clinicaldatasec$DSS.time/365

ROC<-timeROC(T=clinicaldatasec$DSS.time.year,delta=clinicaldatasec$DSS,

marker=clinicaldatasec$score,cause=1,

weighting="marginal",

times=c(predict_3_year,predict_5_year),ROC=TRUE)

pdf("TCGA_ROC_Figure3F.pdf")

plot(ROC,time=predict_3_year,col="#2874C5",title=FALSE,lwd=3)

plot(ROC,time=predict_5_year,col="#C6524A",add=TRUE,title=FALSE,lwd=3)

legend("bottomright",

c(paste("AUC of 3 year survival: ",round(ROC$AUC_1[1],3)),

paste("AUC of 5 year survival: ",round(ROC$AUC_2[2],3))),col=c("#2874C5","#C6524A"),lwd=3)

dev.off()

#################Violin Plots **(Figure 4G-I)**#####

clinicaldata$id <- rownames(clinicaldata)

clinicaldatasec$id <- rownames(clinicaldatasec)

clinicaldata_combinddata<-left_join(clinicaldata,clinicaldatasec,by="id")

clinicaldata_combinddata_sec <- clinicaldata_combinddata %>% dplyr::select(c(1:11,13:16,36,38))

###Stage **(Figure 4G)**

table(clinicaldata_combinddata_sec$ajcc_pathologic_tumor_stage)

clinicaldata_combinddata_sec_stage <- clinicaldata_combinddata_sec[-which(clinicaldata_combinddata_sec$ajcc_pathologic_tumor_stage=="[Not Available]"),]

compare_means(score~ ajcc_pathologic_tumor_stage, data = clinicaldata_combinddata_sec_stage)

my_comparisons<-list( c("Stage I", "Stage IV"),

c("Stage II", "Stage III"),

c("Stage II", "Stage IV"))

pdf("TCGA_Figure4G.pdf",width=4.9,height=5,file = "D:/TCGA_Figure4G.pdf")

ggplot(clinicaldata_combinddata_sec_stage,

aes(x=ajcc_pathologic_tumor_stage,y=score,fill=ajcc_pathologic_tumor_stage)) +

geom_jitter(aes(color=ajcc_pathologic_tumor_stage),alpha=0.5) + scale_color_brewer(palette = 'Set1',direction=-1) +

geom_violin(alpha=0,size=1.4) + theme_bw() +

ggtitle("TCGA cohort")+theme_bw()+ theme(legend.position="none", axis.text.x=element_text(colour="black",family="Times",size=14), axis.text.y=element_text(family="Times",size=14,face="plain"), axis.title.y=element_text(family="Times",size = 14,face="plain"), axis.title.x=element_text(family="Times",size = 14,face="plain"), plot.title = element_text(family="Times",size=15,face="bold",hjust = 0.5),panel.grid.major = element_blank(), panel.grid.minor = element_blank())+

ylab("Risk score")+xlab("") +

stat_compare_means(comparisons = my_comparisons)+

stat_compare_means(label.y = 5,label.x = 1)

dev.off()

###grade **(Figure 4H)**

table(clinicaldata_combinddata_sec$histological_grade)

clinicaldata_combinddata_sec_grade <- clinicaldata_combinddata_sec[-which(clinicaldata_combinddata_sec$histological_grade=="[Unknown]"),]

clinicaldata_combinddata_sec_grade$histological_grade<-as.factor(clinicaldata_combinddata_sec_grade$histological_grade)

clinicaldata_combinddata_sec_grade$histological_grade<- factor(clinicaldata_combinddata_sec_grade$histological_grade,levels = c("Low Grade","High Grade"))

compare_means(score~ histological_grade, data = clinicaldata_combinddata_sec_grade)

my_comparisons<-list( c("Low Grade", "High Grade"))

pdf("TCGA_Figure4H.pdf",width=4.9,height=5,file = "D:/TCGA_Figure4H.pdf")

ggplot(clinicaldata_combinddata_sec_grade,

aes(x=histological_grade,y=score,fill=histological_grade)) +

geom_jitter(aes(color=histological_grade),alpha=0.5) + scale_color_brewer(palette = 'Set1',direction=-1) +

geom_violin(alpha=0,size=1.4) + theme_bw() +

ggtitle("TCGA cohort")+theme_bw()+ theme(legend.position="none", axis.text.x=element_text(colour="black",family="Times",size=14), axis.text.y=element_text(family="Times",size=14,face="plain"), axis.title.y=element_text(family="Times",size = 14,face="plain"), axis.title.x=element_text(family="Times",size = 14,face="plain"), plot.title = element_text(family="Times",size=15,face="bold",hjust = 0.5),panel.grid.major = element_blank(), panel.grid.minor = element_blank())+

ylab("Risk score")+xlab("") +

stat_compare_means(comparisons = my_comparisons)+

stat_compare_means(label.y = 5,label.x = 1.5)

dev.off()

###Progression **(Figure 4I)**

table(clinicaldata_combinddata_sec$PFI)

clinicaldata_combinddata_sec$PFI.1<-ifelse(clinicaldata_combinddata_sec$PFI==1,"Progression","No progression")

clinicaldata_combinddata_sec$PFI.1<-as.factor(clinicaldata_combinddata_sec$PFI.1)

clinicaldata_combinddata_sec$PFI.1<- factor(clinicaldata_combinddata_sec$PFI.1,levels = c("No progression","Progression"))

compare_means(score~ PFI.1, data = clinicaldata_combinddata_sec)

my_comparisons<-list( c("No progression", "Progression"))

pdf("TCGA_Figure4I.pdf",width=4.9,height=5,file = "D:/TCGA_Figure4I.pdf")

ggplot(clinicaldata_combinddata_sec,

aes(x=PFI.1,y=score,fill=PFI.1)) +

geom_jitter(aes(color=PFI.1),alpha=0.5) + scale_color_brewer(palette = 'Set1',direction=-1) +

geom_violin(alpha=0,size=1.4) + theme_bw() +

ggtitle("TCGA cohort")+theme_bw()+ theme(legend.position="none", axis.text.x=element_text(colour="black",family="Times",size=14), axis.text.y=element_text(family="Times",size=14,face="plain"), axis.title.y=element_text(family="Times",size = 14,face="plain"), axis.title.x=element_text(family="Times",size = 14,face="plain"), plot.title = element_text(family="Times",size=15,face="bold",hjust = 0.5),panel.grid.major = element_blank(), panel.grid.minor = element_blank())+

ylab("Risk score")+xlab("") +

stat_compare_means(comparisons = my_comparisons)+

stat_compare_means(label.y = 5,label.x = 1.5)

dev.off()
