# Supplementary material for: Development and Validation of a Six-Gene Prognostic Signature for Bladder Cancer
Source: Front Genet. 2021 Dec 6;12:758612. doi: 10.3389/fgene.2021.758612 (PMC8685517; doi:10.3389/fgene.2021.758612)
Supplement: Supplementary file 2 [file DataSheet1.DOCX]

Supplementary Material

# Supplementary Table

**Supplementary Table 1.** Primer sequences of GAPDH and six signatures (***CDK4, E2F7, COL11A1, BDKRB2, YIF1B and ZNF415***).

| Gene | Primer Sequence |
| --- | --- |
| GAPDH | F:5'-CTGGGCTACACTGAGCACC-3'; |
|  | R:5'-AAGTGGTCGTTGAGGGCAATG-3'; |
| CDK4 | F:5'-ATGGCTACCTCTCGATATGAGC-3'; |
|  | R:5'-CATTGGGGACTCTCACACTCT-3'; |
| E2F7 | F:5'-AATGCAGTGGTTGTTTCTGT-3'; |
|  | R:5'-TGCCATTGCTTCTTCACTAC-3'; |
| COL11A1 | F:5'-TGGTGATCAGAATCAGAAGTTCG-3'; |
|  | R:5'-AGGAGAGTTGAGAATTGGGAATC-3'; |
| BDKRB2 | F:5'-CCGAAAGAAGTCTTGGGAGGT-3'; |
|  | R:5'-CTGGCGTTCCACGGAGATG-3'; |
| YIF1B | F:5'-GCTGTGGACACCATGTATGTG-3'; |
|  | R:5'-CAGCCACCAAAACGTAGGTGA-3'; |
| ZNF415 | F:5'-TGCCTGAACTCTACACAGAGG-3'; |
|  | R:5'-AGTTACGAGACAGATCCAGGG-3'. |
